# Supplementary material for: Enhanced Depolymerization of Actin Filaments by ADF/Cofilin and Monomer Funneling by Capping Protein Cooperate to Accelerate Barbed-End Growth
Source: Curr Biol. 2017 Jul 10;27(13):1990–1998.e5. doi: 10.1016/j.cub.2017.05.036 (PMC5505869; doi:10.1016/j.cub.2017.05.036)
Supplement: Document S1. Figures S1 and S2 [file mmc1.pdf]

Current Biology, Volume *27*

Supplemental Information

Enhanced Depolymerization of Actin Filaments by ADF/Cofilin and Monomer Funneling by Capping Protein Cooperate to Accelerate Barbed-End Growth

Shashank Shekhar and Marie-France Carlier

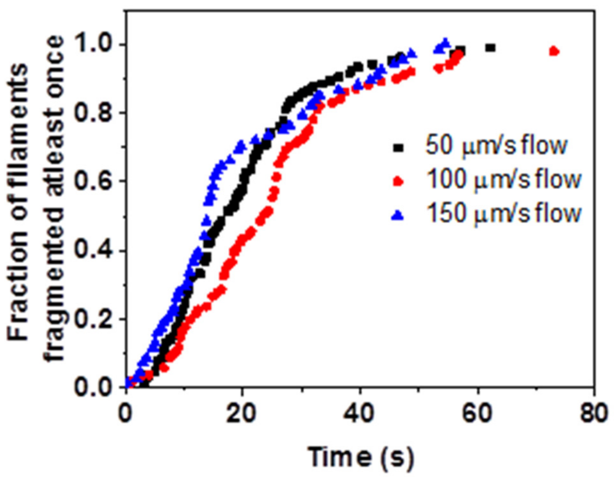

Figure S1. ADF-induced fragmentation is not affected by flow (in range of flow-rates used here). Related to Figure 1.

Frequency of ADF-induced fragmentation (at 5 µM ADF) was seen to be independent of the flow-rate in the range 50 µM/s to 150 µM/s flows (n = 100 filaments). ADF-induced depolymerization and fragmentation measurements were carried out at around 50 µM/s flow rates.

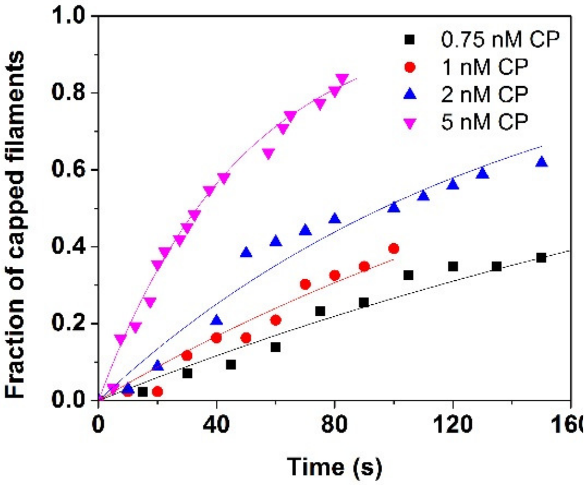

A

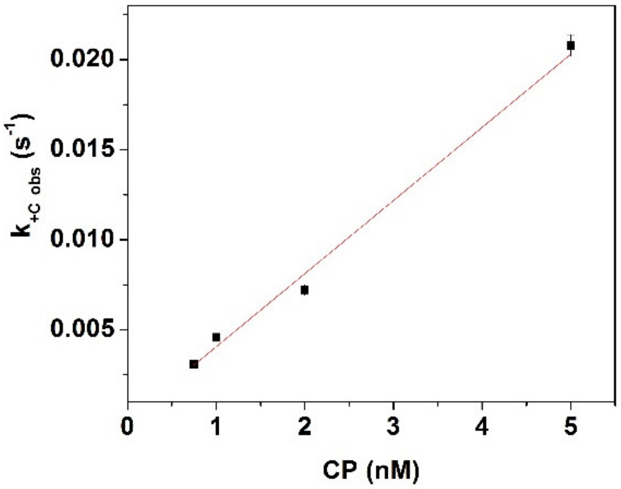

B

Figure S2. Measurement of the association rate constant of CP to the barbed end (k_{+C}). Related to Figure 3.

(A) Measurement of association rate constant for CP for tracer barbed end. Increase in fraction of barbed ends being capped by Capping Protein (B + C → BC) in the presence of 0.75 nM (black), 1 nM (red), 2 nM (blue) and 5 nM (magenta) CP. Symbols are actual data and lines are exponential fits (n = 30-40 filaments). (**B**) Linear fit of the relationship between observed on-rate of CP on CP concentration gives k_{+C} = 4.06± 0.17 $\mu M^{-1}.s^{-1}$.
