# Supplementary figures and images for: Enhanced Depolymerization of Actin Filaments by ADF/Cofilin and Monomer Funneling by Capping Protein Cooperate to Accelerate Barbed-End Growth
Source: Curr Biol. 2017 Jul 10;27(13):1990–1998.e5. doi: 10.1016/j.cub.2017.05.036 (PMC5505869; doi:10.1016/j.cub.2017.05.036)

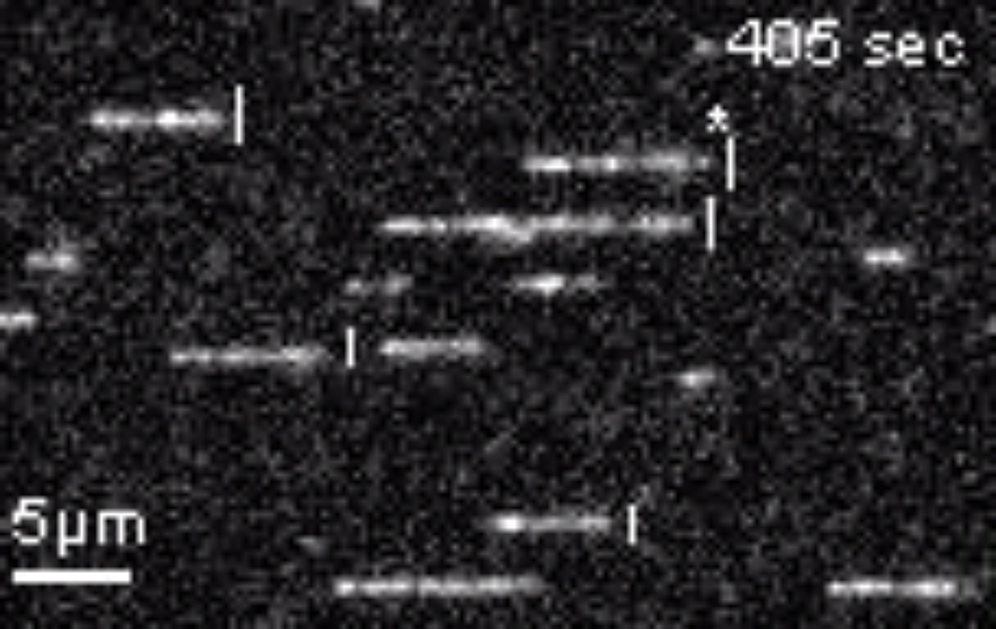

Supplement: Movie S1. Enhanced Pointed-End Filament Depolymerization by 0.1 μM ADF, Related to Figure 1C — Filaments were grown from coverslip-anchored gelsolin-actin complexes. The barbed ends were bound to anchored Gelsolin and the pointed ends were free in the flow. The filaments were then aged to ADP-F-actin by exposing to F-buffer for 15 minutes. Free pointed ends of these filaments were then exposed to 0.1 μM ADF in F buffer. Filaments show fast depolymerize from their pointed ends as well as fragmentation. “White asterisks” indicate the depolymerizing pointed ends of a few select filaments that depolymerize for periods long enough to observe depolymerization without fragmentation. Short vertical white lines represent the initial location of pointed ends at t=0. [file mmc2.jpg]

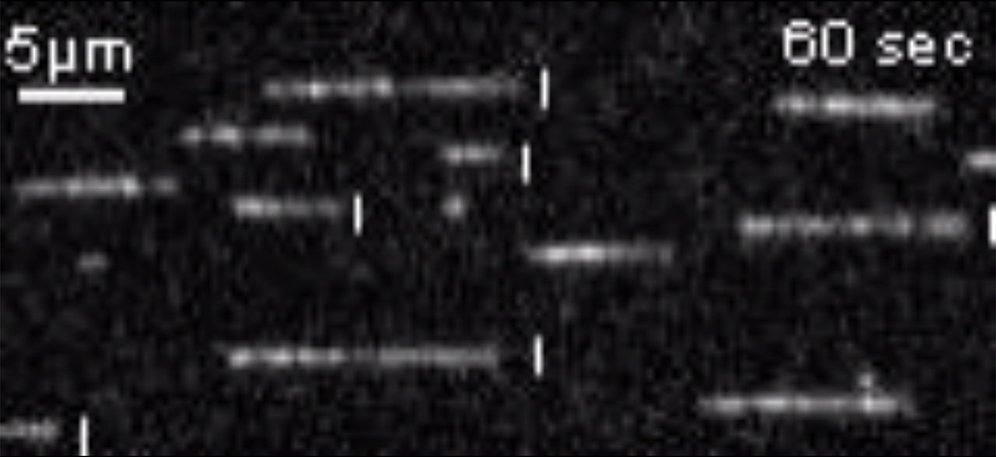

Supplement: Movie S2. Enhanced Pointed-End Filament Depolymerization by 2.5 μM ADF, Related to Figure 1C — Filaments were grown from coverslip-anchored gelsolin-actin complexes. The barbed ends were bound to anchored Gelsolin and the pointed ends were free in the flow. The filaments were then aged to ADP-F-actin by exposing to F-buffer for 15 minutes. Free pointed ends of these filaments were then exposed to 2.5 μM ADF in F buffer. Filaments show fast depolymerize from their pointed ends as well as fragmentation. “White asterisks” indicate the depolymerizing pointed ends of a few select filaments that depolymerize for periods long enough to observe depolymerization without fragmentation. Short vertical white lines represent the initial location of pointed ends at t=0. [file mmc3.jpg]

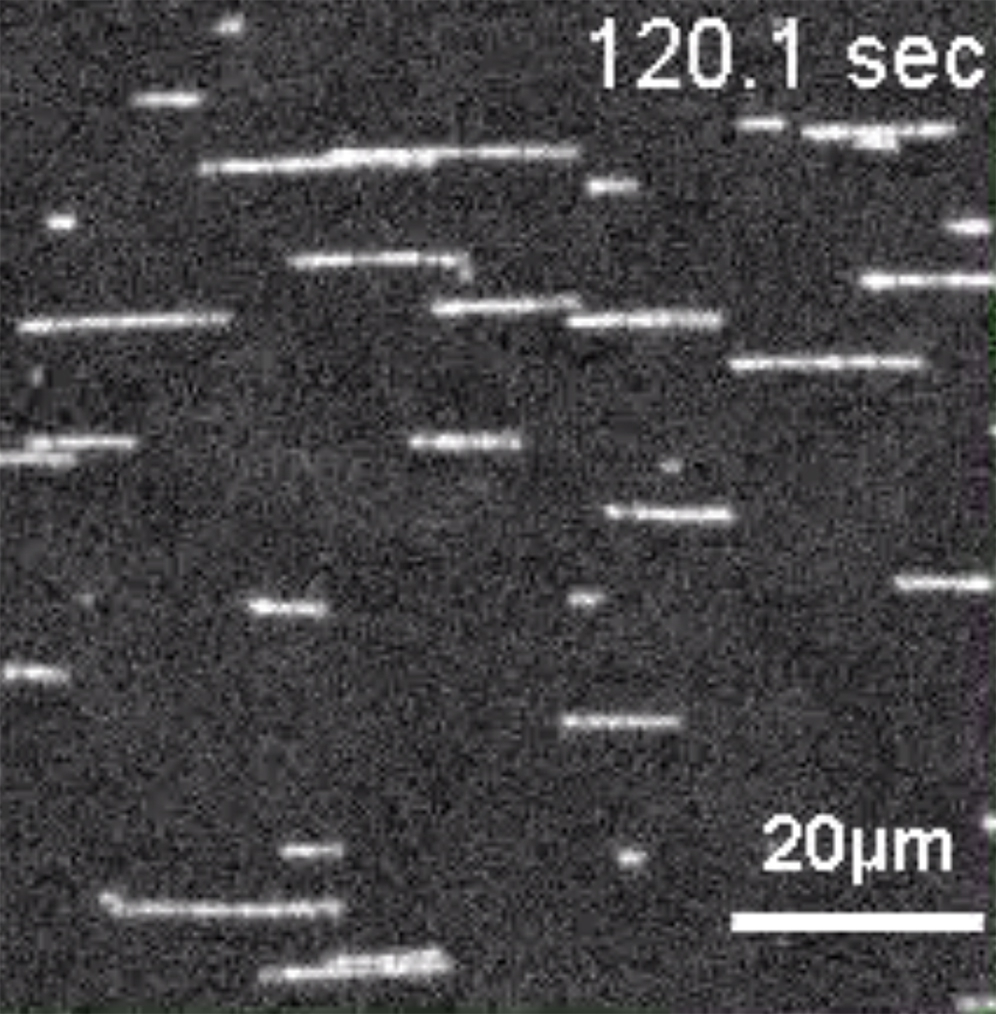

Supplement: Movie S3. Concentration-Dependent Enhancement of Filament Barbed-End Depolymerization by ADF, Related to Figure 1D — Filaments were grown from coverslip-anchored spectrin-actin seeds with filament barbed ends free in the flow. The filaments were then aged to ADP-F-actin by exposing to F-buffer for 15 minutes. Free barbed ends of these filaments were then exposed to 2 μM ADF in F buffer. Filaments show fast depolymerization from their barbed ends as well as fragmentation. [file mmc4.jpg]

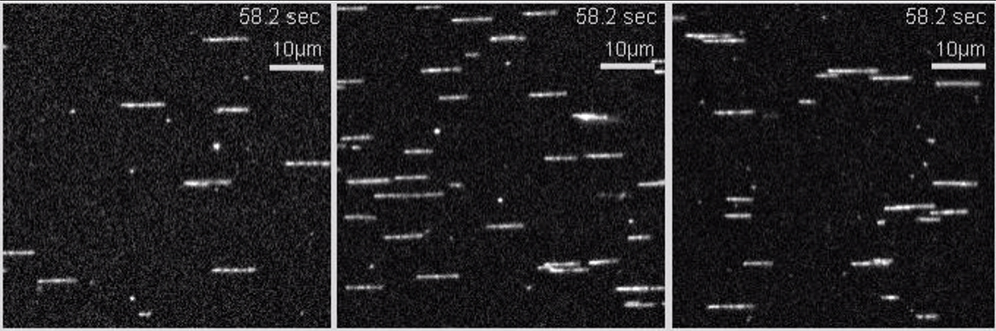

Supplement: Movie S4. Concentration-Dependent Enhancement of Filament Severing by ADF, Related to Figure 1E — Montage of time-lapsed images of effect of ADF filament severing. Filaments were grown from spectrin-actin seeds anchored on the coverslip to a length of about 10μm. The filaments were then aged to ADP-F-actin by exposing to F-buffer for 15 minutes in presence of Capping Protein (10 nM, to prevent free barbed end depolymerization). ADP-F-actin filaments were then exposed to solution containing 1 μM ADF in F buffer (left) and 3.5 μM ADF in F buffer (right). 10nM CP was present throughout to ensure that filament length changed due to severing only (and not due to depolymerization of barbed ends). Filament fragmentation increases with ADF concentration. [file mmc5.jpg]

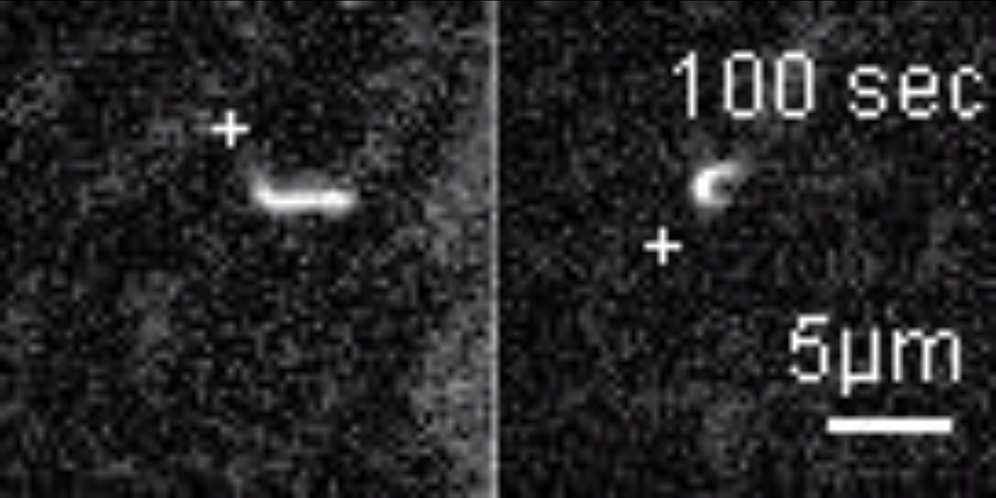

Supplement: Movie S5. Filament Treadmilling in Presence of ADF, Related to Figure 2D — Montage of two separate filaments from the flow (0.75 μM ADF) which incidentally got stuck to the coverslip surface. These filaments with both their ends free, are seen to treadmill with equivalent elongation of their barbed ends (denoted by “+”) and shrinkage of their pointed ends. Constant filament length is maintained during treadmilling. Filament on the right is also seen to fragment towards the end of the movie. [file mmc6.jpg]
