# Supplementary material for: Enhanced Depolymerization of Actin Filaments by ADF/Cofilin and Monomer Funneling by Capping Protein Cooperate to Accelerate Barbed-End Growth
Source: Curr Biol. 2017 Jul 10;27(13):1990–1998.e5. doi: 10.1016/j.cub.2017.05.036 (PMC5505869; doi:10.1016/j.cub.2017.05.036)
Supplement: Document S2. Article plus Supplemental Information [file mmc7.pdf]

Report

Current Biology

Enhanced Depolymerization of Actin Filaments by ADF/Cofilin and Monomer Funneling by Capping Protein Cooperate to Accelerate Barbed-End Growth

Graphical Abstract

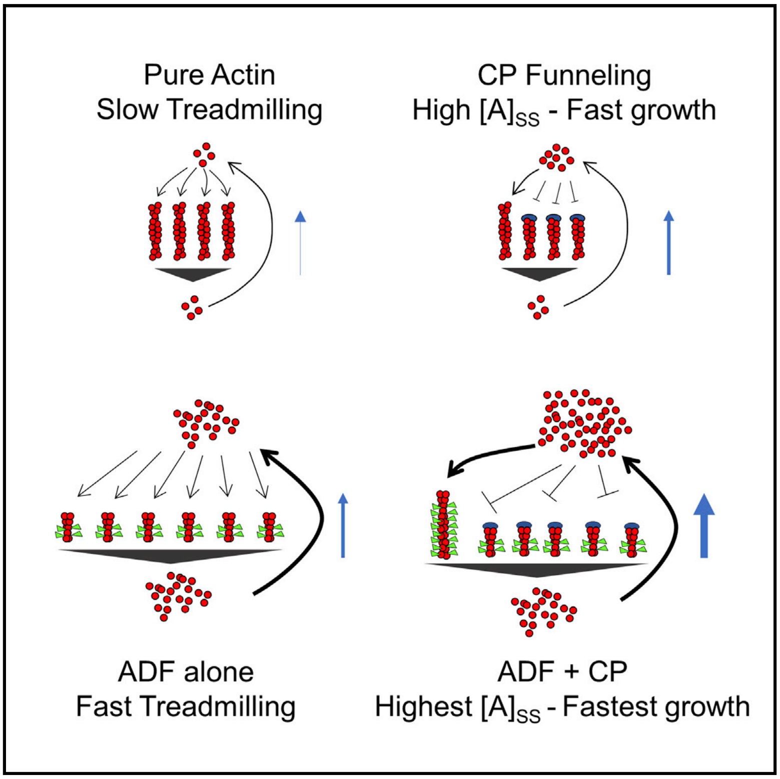

Highlights

- ADF enhances barbed- and pointed-end depolymerization of actin filaments
- Capping protein funnels monomers from all pointed ends to the few non-capped barbed ends
- ADF and capping protein synergy leads to skyrocketing of filament elongation rates

Authors

Shashank Shekhar, Marie-France Carlier

Correspondence

shashank.shekhar@yahoo.com (S.S.), mfactin@gmail.com (M.-F.C.)

In Brief

Shekhar and Carlier provide the first direct visual evidence of rapid depolymerization of actin filaments by actin depolymerizing factor as well as of the long-hypothesized monomer funneling by capping protein. Using a novel microfluidics approach, they also reveal how the two proteins synergize to produce skyrocketing of filament growth rates.

Shekhar & Carlier, 2017, Current Biology *27*, 1990–1998
July 10, 2017 © 2017 The Author(s). Published by Elsevier Ltd.
http://dx.doi.org/10.1016/j.cub.2017.05.036

CellPress

Current Biology
Report

Enhanced Depolymerization of Actin Filaments by ADF/Cofilin and Monomer Funneling by Capping Protein Cooperate to Accelerate Barbed-End Growth

Shashank Shekhar[1,2,*] and Marie-France Carlier[1,*]
[1]Cytoskeleton Dynamics and Cell Motility, Institute for Integrative Biology of the Cell (I2BC), CEA, CNRS, Université Paris-Sud, Université Paris Saclay, 91198 Gif-sur-Yvette Cedex, France
[2]Lead Contact
*Correspondence: shashank.shekhar@yahoo.com (S.S.), mfactin@gmail.com (M.-F.C.)
http://dx.doi.org/10.1016/j.cub.2017.05.036

SUMMARY

A living cell's ability to assemble actin filaments in intracellular motile processes is directly dependent on the availability of polymerizable actin monomers, which feed polarized filament growth [1, 2]. Continued generation of the monomer pool by filament disassembly is therefore crucial. Disassemblers like actin depolymerizing factor (ADF)/cofilin and filament cappers like capping protein (CP) are essential agonists of motility [3–8], but the exact molecular mechanisms by which they accelerate actin polymerization at the leading edge and filament turnover has been debated for over two decades [9–12]. Whereas filament fragmentation by ADF/cofilin has long been demonstrated by total internal reflection fluorescence (TIRF) [13, 14], filament depolymerization was only inferred from bulk solution assays [15]. Using microfluidics-assisted TIRF microscopy, we provide the first direct visual evidence of ADF's simultaneous severing and rapid depolymerization of individual filaments. Using a conceptually novel assay to directly visualize ADF's effect on a population of pre-assembled filaments, we demonstrate how ADF's enhanced pointed-end depolymerization causes an increase in polymerizable actin monomers, thus promoting faster barbed-end growth. We further reveal that ADF-enhanced depolymerization synergizes with CP's long-predicted "monomer funneling" [16] and leads to skyrocketing of filament growth rates, close to estimated lamellipodial rates. The "funneling model" hypothesized, on thermodynamic grounds, that at high enough extent of capping, the few non-capped filaments transiently grow much faster [15], an effect proposed to be very important for motility. We provide the first direct microscopic evidence of monomer funneling at the scale of individual filaments. These results significantly enhance our understanding of the turnover of cellular actin networks.

RESULTS AND DISCUSSION

Force is produced against membranes by filament barbed-end assembly, which requires maintenance of a pool of polymerizable actin monomers feeding growing barbed ends. The steady-state monomer pool is maintained by pointed-end filament depolymerization. In this treadmilling concept, the bulk rate of barbed-end growth of the filament population balances the rate of pointed-end depolymerization. Pure actin filaments treadmill very slowly [17] compared to the rapid apparent treadmilling rates observed in intracellular lamellipodial actin arrays; hence, cellular upregulation of the intrinsically slow treadmilling is required [18].

Actin depolymerizing factor (ADF)/cofilin and capping protein (CP) play a key role in motility in vivo [4–8] and in reconstituted in vitro assays [19–21]. However, the molecular mechanisms underlying how ADF by itself enhances motility are still unclear. In most single-filament studies, ADF/cofilin has been found to only fragment actin filaments [13, 14, 22, 23]. However, severing (in absence of barbed-end capping) cannot increase the steady-state monomer concentration. Severing identically increases the number of barbed and pointed ends; all individual filaments therefore treadmill at an unchanged rate, and the size of the monomer pool remains unchanged [1]. One group has however proposed that ADF's enhanced filament depolymerization, monitored by bulk solution light scattering, was responsible for ADF's effect on motility [15, 24, 25]. This proposal has been questioned because ADF's enhanced filament depolymerization has so far never been microscopically visualized. Disassembly by ADF has rather been attributed to shortening by fragmentation [14]. In the present study, we provide the first visual evidence of ADF's filament depolymerization in addition to its well-characterized filament severing.

CP has also been shown to enhance motility in a concentration-dependent fashion [19, 26–28]. When a large number of filaments are exposed to CP, the fraction of filaments getting capped will be dependent on CP concentration (fraction capped, $\gamma = ([CP]/[CP] + K_C)$, where $K_C = 0.1$ nM is the equilibrium dissociation constant for CP binding to barbed ends) [29]. When an increasing fraction of filaments gets capped, monomers released from all pointed ends feed the growth of only a few barbed ends that are not capped. These few non-capped barbed ends individually grow much faster than if all barbed

 © 2017 The Author(s). Published by Elsevier Ltd.
This is an open access article under the CC BY license (http://creativecommons.org/licenses/by/4.0/).

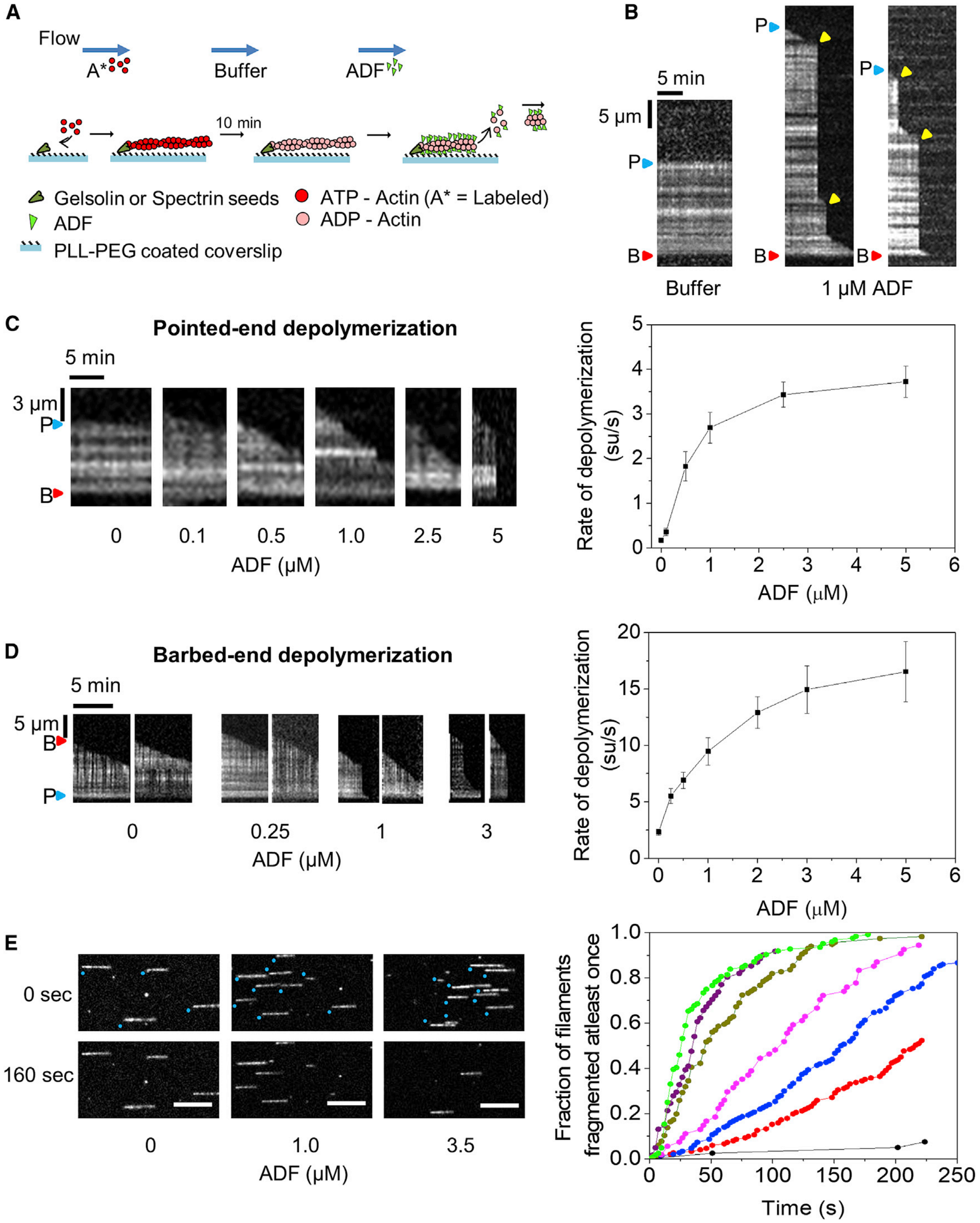

Figure 1. ADF Severs Filaments and Enhances Depolymerization at Filament Pointed and Barbed Ends

(A) Schematics of the setup for single-filament depolymerization. Filaments exposing free pointed ends or barbed ends were grown from coverslip-anchored gelsolin-actin complexes or spectrin-actin seeds, respectively, and sequentially exposed to F-buffer and ADF in F-buffer.

(B) Representative kymographs of ADP-F-actin filaments anchored at barbed ends (B, red triangle) and exposing free pointed ends (P, blue triangle) to either F buffer only (left panel) or 1 µM ADF in F buffer (two right panels). Severing events, yellow triangles.

(C) ADF concentration dependence of pointed-end depolymerization. Left: representative kymographs. Right: rate of pointed-end depolymerization versus ADF (see Movies S1 and S2).

(D) ADF enhances depolymerization at filament barbed ends. Left: representative kymographs. Right: barbed-end depolymerization rate versus ADF concentration (see Movie S3). Error bars represent SD; each data point represents the average of 50 measurements cumulated from three independent experiments. All depolymerization rates were found to be statistically significant ($p < 0.05$; two-sample t test) compared to the control (0 ADF).

(legend continued on next page)

ends were free to grow (referred to as the ''funneling effect''; Figure 4B) [16]. This model has been contested, and CP has instead been proposed to enhance nucleation [12].

So far, ADF's enhanced depolymerization and CP's funneling have only been interpreted from bulk solution experiments. Nevertheless, ADF in association with its effector Aip1 appears to cause enhanced single-filament disassembly [30, 31]. Here, we use microfluidics-assisted single-filament imaging [32, 33] to reveal both severing and rapid depolymerization of actin filaments by ADF alone. This assay is then adapted to monitor the effects of ADF and CP (alone and together) on the concentration of ATP monomers co-existing with filaments at steady state, thus providing first direct visual evidence of the funneling effect. Filaments are anchored at one of their ends and not tethered to the surface all along their length. They are then exposed to a microfluidic flow of known biochemical composition. This method therefore avoids any mechanical interference with the change in filament twist due to ADF binding and also does not alter competition between side-binding anchoring proteins and ADF. Results presented here provide the first single-filament demonstration that ADF and CP work synergistically to enhance treadmilling of individual filaments, accounting for their in vivo function.

ADF Enhances Depolymerization Rate of Pointed and Barbed Ends

We first studied the effect of ADF on filament pointed ends. Filaments initiated from surface-anchored gelsolin-actin complexes were allowed to elongate at pointed ends. Filaments were then aged for 10 min to complete the transition to ADP-F-actin. This period is 6-fold the Pi release half-time ($t_{1/2}$ = 102 s) [34]. ADP filaments were then exposed to a flow of polymerization buffer (F-buffer: 5 mM Tris-HCl [pH 7.8], 2 mM ATP, 1 mM $MgCl_2$, 0.2 mM EGTA, 50 mM KCl, 10 mM DTT, and 1 mM DABCO) containing increasing amounts of human ADF (Figure 1A). Filaments exposed to F-buffer alone expectedly did not appreciably fragment within the observation period of 10 min and depolymerized extremely slowly from the pointed end (0.17 ± 0.04 subunits/s; SD; n = 50 filaments; three independent experiments), in agreement with earlier single-filament studies (0.16 su/s) [35]. These values are slightly lower than those from bulk measurements [17], possibly due to the slower dissociation of photo-induced F-actin dimers [32]. Upon exposure to ADF, we observed both filament severing as well as faster pointed-end depolymerization (between consecutive severing events; Figure 1B). The frequency of severing increased with ADF concentration, as seen previously [22, 36]. Figure 1E shows that 50% of filaments (mean length = 10 μm) have severed at least once at $t_{1/2}$ = 40 s in the presence of 5 μM ADF, consistent with the scheme F → 2F and a first-order fragmentation rate constant of $\ln(2)/t_{1/2}$ = 0.017 s^{-1} for a 10-μm-long filament (see Movie S4). The rate of pointed-end depolymerization also increased with ADF concentration in a saturating fashion up to a maximal rate of 3.72 ± 0.35 su/s (n = 50 filaments; three independent experiments) at 5 μM ADF (Figure 1C; Movies S1 and S2). The 22-fold increase in depolymerization rate at pointed ends is similar to the 25-fold increase observed in bulk measurements [15].

To evaluate the effect of ADF on depolymerization at barbed ends, we grew filaments from coverslip-anchored spectrin-actin seeds (Figure 1A). The free barbed ends of the aged ADP-F-actin filaments were then exposed to ADF in F-buffer as above. In buffer alone, barbed ends depolymerize at 2.31 ± 0.27 su/s, similar to previous reports of ~1.4 su/s [35, 37]. The barbed-end depolymerization rate increased with ADF concentration in a saturating fashion (Figure 1D) up to about 16.54 ± 2.68 su/s at 5 μM ADF (n = 50 filaments; three independent experiments), in a trend similar to pointed ends (Figure 1C). High frame rate imaging (2.45 s per frame) ensured that the observed rate of depolymerization was not just a collection of short severing events (see Movie S3).

ADF's destabilization of actin-actin interactions in the filament [38], which promotes filament severing [13, 22, 23, 39], might also cause the enhanced disassembly at filaments ends. Why has this not been seen earlier? A possible explanation lies in the methods used in earlier studies and maybe in the acknowledged quantitative differences between the various cofilins and ADF variants [40, 41]. In previous studies, filaments were mostly maintained in the evanescent field by surface anchoring [13, 22]. The filament fragments generated by severing immediately left the evanescent field [22], and shortening of the anchored filaments was interpreted as disassembly [14]. Our method, in which the flow (50–100 μm/s) maintains the filaments aligned in the evanescent field, allows visualization and accurate measurement of both severing and depolymerization. Earlier work from our lab used this technique to apply controlled forces on actin filaments anchored at one end (see Figure 1A for experimental schematic) [42]. Whereas the applied drag force increases steadily toward the anchorage point, the force is negligible at the free end. Low-flow rates as used in this study (50–100 μm/s) do not affect elongation, depolymerization [34], or protein activity at the free end [42]. ADF-induced depolymerization at the free end will therefore be unaffected. We further checked that flow had no effect on ADF-induced severing in the range of flow rates used here (see Figure S1). Future experiments with fluorescently labeled ADF will be valuable in confirming the results presented here. However, this has so far been a challenge because many of ADF's eight cysteine residues are close to its actin-interaction regions [22].

Twinfilin and Srv2 together have recently been found to promote a similar increase in pointed-end (17-fold) and barbed-end (3-fold) depolymerization [37]. Aip1 also enhances depolymerization of ADF/cofilin-decorated filaments [31, 43]. However, enhanced depolymerization by ADF/cofilin alone has so far never been visualized. We have demonstrated that ADF alone is sufficient for both fragmentation and depolymerization of filaments, with identical concentration dependence. Both properties thus reflect the lower structural [38], mechanical [44], and thermodynamic stability of ADF-decorated than bare filaments. We do not see the reported decrease in severing of cofilin at high concentrations [13]. The

(E) Time-lapsed images of ADP-F-actin filaments with their pointed ends anchored (blue dots) and barbed ends capped being severed by ADF (flow direction: left to right) (see Movie S4; Figure S1) (left) and cumulative distribution function of the first severing event per filament as a function of time for different ADF concentrations—no ADF (black), 0.5 μM (red), 1 μM (blue), 2 μM (magenta), 3.5 μM (dark green), 5 μM (purple), and 10 μM (light green). Each curve consists of about 70–80 filaments of average length around 10 μm.

severing mechanism used by ADF differs from the "hit and cut" mechanism used by gelsolin, Spire, or Cobl.

ADF Increases Steady-State Monomer Concentration

As discussed previously (see [1] for a detailed quantitative discussion), severing of pure actin filaments by itself does not change the steady-state monomer concentration (~0.1 μM). ADF's effect on G-actin concentration has been evaluated by bulk assays—SDS-PAGE of supernatants of high-speed centrifuged F-actin or measurements of ADF-increased amount of ATP-G-actin, amplified by sequestration by thymosin β4 [24].

We designed a novel microfluidics-assisted total internal reflection fluorescence microscopy (TIRFM) assay to directly measure the monomeric ATP-actin concentration and monitor how ADF affects it. Tracer filaments exposing free barbed ends were then elongated from immobilized spectrin-actin seeds in a flow containing 1 μM G-actin (10% Alexa 488 labeled) and 4 μM profilin (see Figure 2A for a schematic). The tracer filaments were then exposed to a flow containing F-buffer with 5 μM 10% Alexa-488-labeled F-actin, 3 μM profilin, and varying amounts of ADF incubated to reach steady state (~2 hr) [15]. Profilin enhances efficiency of treadmilling by preventing monomer association to pointed ends. The elongation rate of tracer filaments acts as a reliable reporter of the concentration of polymerizable monomers (mainly profilin-actin; see STAR Methods) co-existing with the population of flowing filaments at steady state. By varying ADF concentration, the effect of ADF on steady-state monomer concentration was quantified (Figure 2B).

In absence of ADF, tracer barbed ends grew very slowly (0.14 ± 0.05 su/s; close to the rate of pointed-end disassembly and consistent with 0.1 μM profilin-actin in the mix). In presence of ADF, tracer barbed ends grew faster, up to about 4 su/s at 2 μM ADF, which translates into a concentration of 0.54 μM profilin-actin (and negligible G-actin; Figure 2B). The decline at higher ADF most likely reflects ADF-mediated inhibition of conversion of ADF-ADP-G-actin to ATP-G-actin [15]. ADF-induced severing events are visible between periods of barbed-end growth (Figure 2C). Interestingly, a few filaments from the bulk flow sometimes landed and bound non-specifically to the glass surface, exposing their two ends to the flow. Because they were part of the bulk flow, these filaments were actually at steady state. They display barbed-end growth and pointed-end shrinkage at identical rates, maintaining constant length, i.e., treadmilling (Figures 2D and 2E; Movie S5). In conclusion, ADF enhances treadmilling by establishing a higher steady-state monomer concentration.

CP Funnels Monomers to Uncapped Barbed Ends

Capping of barbed ends is essential for actin-based motility [20, 26, 28]. CP has been proposed to "funnel" actin monomers released from all pointed ends onto a few non-capped filaments, locally initiated in cells by surface-bound nucleators, which transiently grow faster [19, 20]. Funneling is expected to increase with the extent of capping and be most effective between 90% and 99.9% capping [45]. Pending direct visualization, this hypothesis has been challenged [12]. We investigated the "funneling effect" of CP using the above assay.

Tracer barbed ends were exposed to a steady-state solution of 5 μM 10% Alexa-488-labeled F-actin, profilin (3 μM), and varying amounts of CP (incubated overnight in F-buffer to reach steady state). At 0.2 nM CP, when 67% of bulk filaments are capped (Equations 7 and 8; STAR Methods), the tracer barbed ends elongate at 0.53 ± 0.14 su/s, corresponding to a concentration of 130 nM profilin-actin and 5 nM G-actin monomers (Figures 3A and 3B). At 5 nM CP (98% bulk filaments capped), the tracer growth rate of 8.06 ± 0.28 su/s corresponds to a steady-state 0.85 μM profilin-actin and 40 nM G-actin. Expectedly, tracer ends also get capped faster with increasing CP concentration (k_{+C} = 4.06 ± 0.17 $\mu M^{-1}s^{-1}$; Figure S2) [29]. The results demonstrate a non-linear dependence of the steady-state monomer concentration on the extent of capping, notably amplified by profilin (Figure 3B) [16, 45].

At CP concentrations above 5 nM, tracer filaments get capped too rapidly to allow elongation rate measurements. We therefore used formin-bound barbed ends as tracers. The 500-fold lower affinity of CP for formin-bound barbed ends [46, 47] allowed us to test higher CP concentrations in the flowing F-actin mix (up to 20 nM CP; 99.5% capping). Fluorescent filaments were nucleated from surface-anchored formin mDia1 FH1-FH2 by flowing in 1 μM labeled G-actin with 1 μM profilin. These formin-anchored "tracer" filaments were then exposed to a flow containing unlabeled 5 μM F-actin, 3 μM profilin, and varying amounts of CP. The fluorescent distal fragment of the tracer filament moves due to insertional addition of unlabeled actin monomers (bound to profilin) at formin-anchored barbed ends (Figure 3C). The elongation rate of formin-bound tracer ends increased up to 130 su/s at 20 nM CP (Figures 3D and 3E). Assuming a value of 50 $\mu M^{-1}.s^{-1}$ for the association rate constant of profilin-actin to formin-bound filaments [42], the 130 su/s elongation rate translates to a steady-state concentration of profilin-actin of about 2.6 μM (at equilibrium with 0.6 μM ATP-G-actin, close to the critical concentration for pointed end assembly). In conclusion, capping of filaments at steady state enhances the growth rate of the few uncapped filaments by increasing the steady-state concentration of polymerizable monomers, thus validating the "funneling effect."

Results presented here clarify a discrepancy between the reported enhancement of actin-based propulsion of *Listeria* and N-WASP beads by CP, inhibition at high capper concentrations [19, 27], which were at variance with the observations and interpretation of CP function made by Akin and Mullins [12]. A key difference between the experiments of Akin and Mullins and ours is that, whereas our flowing actin solutions consist of F-actin (and actin monomers) at steady state, Akin and Mullins's experiments contained only G-actin and CP at time zero. Hence, the filament elongation rate and resulting propulsion rates varied as G-actin was depleted with time. A recent theoretical study argued that CP increases monomer concentration only near the location of branched nucleation at the surface of an ActA-coated bead [11]. In contrast, our results actually show a global increase in bulk monomer concentration. Thus, our steady-state in vitro results appear to account for in vivo effects of CP on motility [1].

ADF and CP Synergistically Enhance the Concentration of Polymerizable Actin Monomers and Barbed-End Assembly at Steady State

We have thus far shown that ADF and CP individually increase actin monomer concentration. We next explored their functional cooperation when they are present together with F-actin. Tracer barbed ends were nucleated and exposed to solutions of F-actin

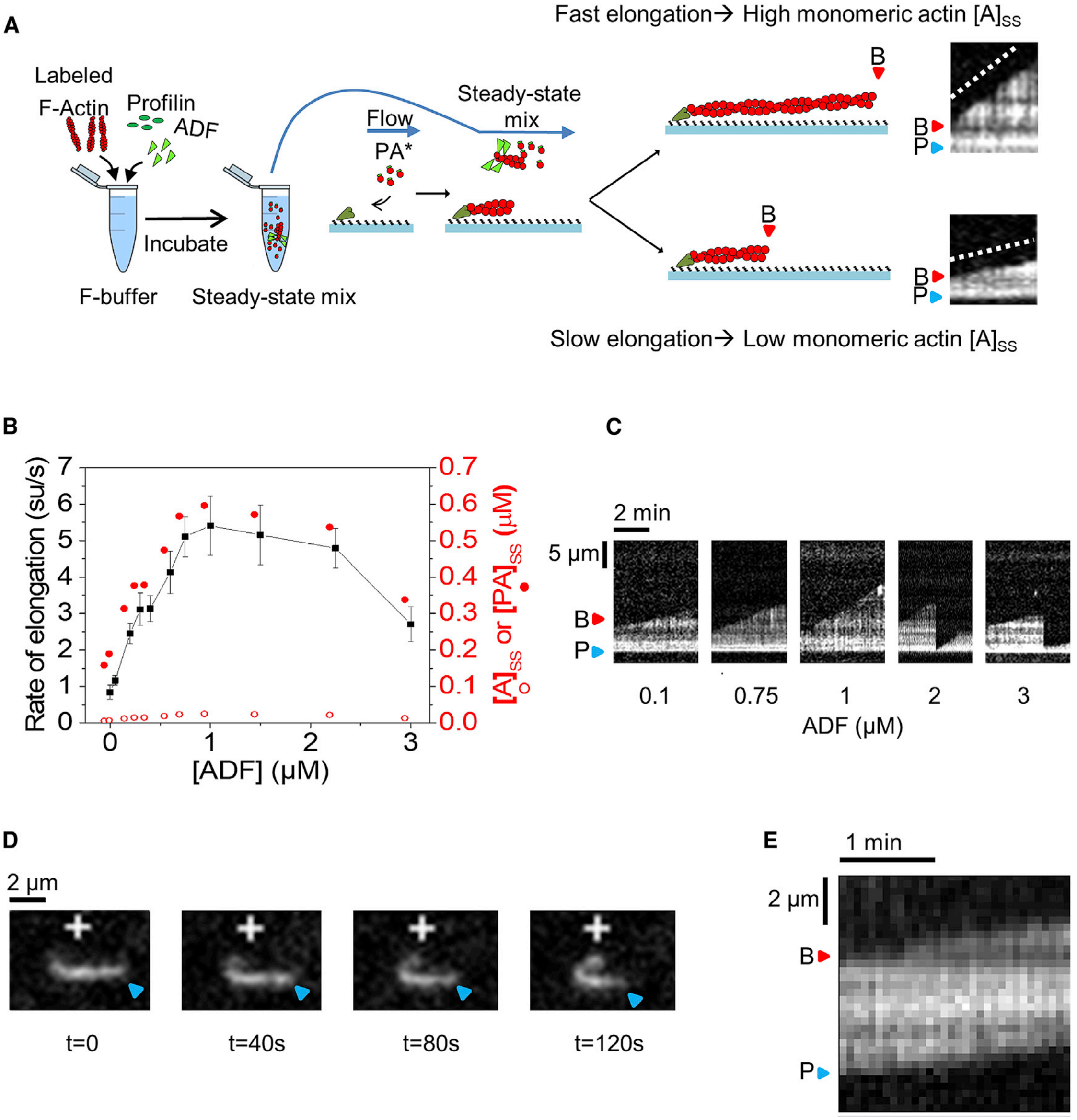

Figure 2. ADF Increases the Steady-State Concentration of Monomeric Actin

(A) Sketch of the setup to measure the amount of steady-state ATP-monomers as a function of ADF. Tracer filament free barbed ends were exposed to solutions of pre-assembled 5 μM 10% Alexa-488-labeled F-actin, 3 μM profilin, and varying amounts of ADF at steady state (see STAR Methods). Example kymographs show fast/slow elongation as indicators of high/low values of the steady-state amount of polymerizable actin actin-monomers ($[A]_{SS}$ + $[PA]_{SS}$). P, blue triangles, pointed ends; B, red triangles, barbed ends.

(B) Elongation rate of tracer barbed ends (black squares and curve) as a function of ADF concentration. Error bars represent SD. Each data point was averaged from at least 50 filaments over three experiments; all elongation rates (except at 50 nM ADF) are statistically significant ($p < 0.05$; two-sample t test) compared to the control (0 ADF). Corresponding steady-state concentrations of actin monomers (profilin-actin $[PA]_{SS}$, red closed circles; free G-actin $[A]_{SS}$, red open circles; see Equations 5 and 6 in STAR Methods).

(C) Representative kymographs.

(D) Filaments from the steady-state flow (0.75 μM ADF), incidentally stuck to the coverslip surface, display barbed-end (+) elongation and pointed-end shrinkage (see Movie S5).

(E) Kymograph showing constant filament length maintained during treadmilling. B and P indicate the two ends.

and profilin at steady state, containing either ADF alone, CP alone, or both (Figures 3F and 3G). At 1 μM ADF and 5 nM CP, the elongation rate of tracer barbed ends was 24 su/s as compared to 4 su/s in presence of 1 μM ADF alone and 8 su/s in presence of 5 nM CP alone. ADF and CP therefore synergize and promote a 160-fold faster elongation rate than in absence of CP and ADF (~0.14 su/s). With 1 μM ADF and 5 nM CP, steady-state monomer concentration increases to about 2.5 μM (Figure 3G). Initial total actin concentration (5 μM) was chosen such that it is higher than the maximal steady-state

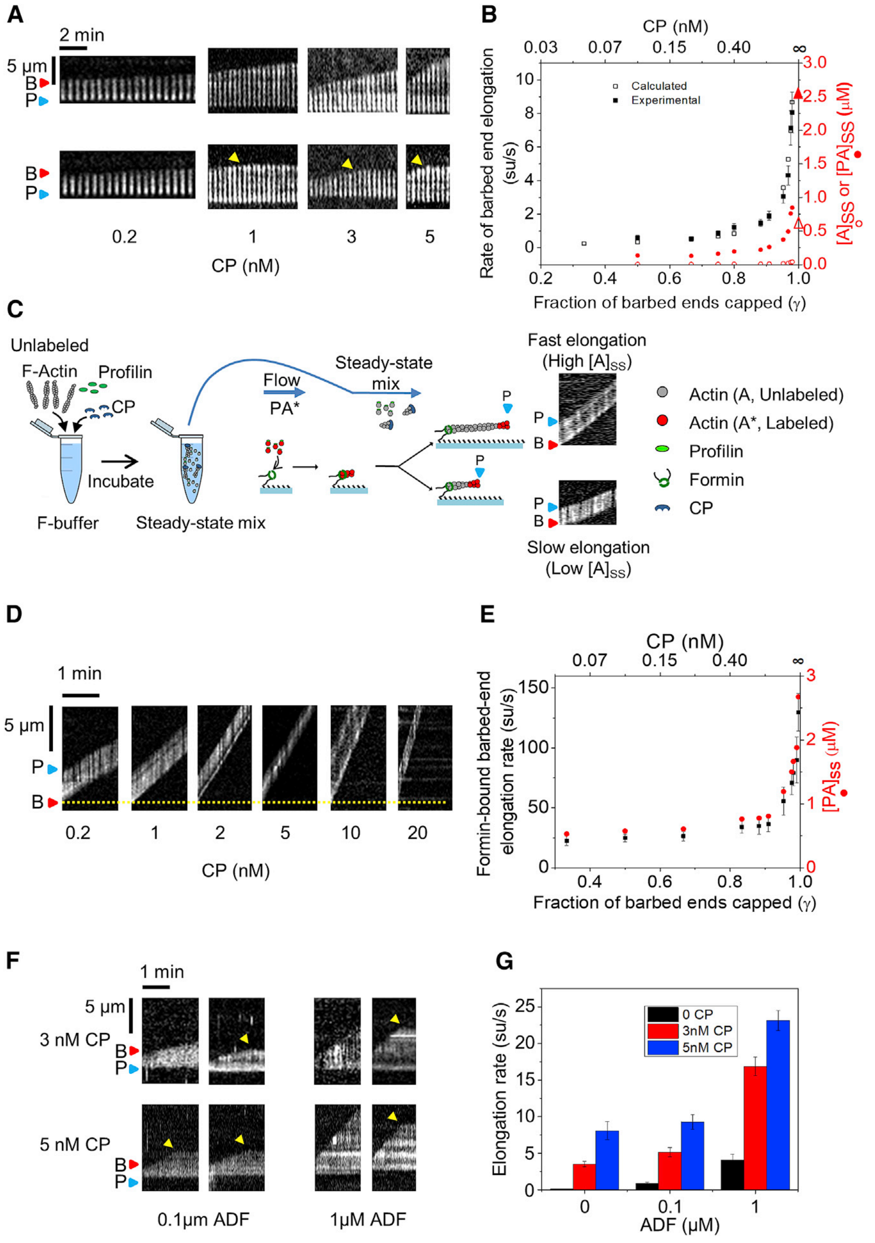

Figure 3. CP Increases Barbed-End Elongation Rate in Synergy with ADF

Experimental conditions are as in Figure 2 except ADF replaced by CP. Tracer barbed ends were exposed to a flow containing pre-assembled 5 μM F-actin (Alexa 488 labeled), 3 μM profilin, and CP.

(A) Representative kymographs of free barbed ends elongating (two for each condition). Arrest of growth by capping is indicated by yellow arrows. P, blue triangles, pointed ends; B, red triangles, barbed ends. See Figure S2.

(B) Rate of barbed-end elongation versus CP concentration: experimental data (black) are plotted versus the calculated rate of tracer elongation (see Equations 7 and 8; STAR Methods) and the corresponding steady-state concentrations of profilin-actin $[PA]_{SS}$ (red closed circles) and free G-actin $[A]_{SS}$ (red open circles; see Equations 5 and 6).The values of $[A]_{SS}$ and $[PA]_{SS}$ at 100% capping are indicated as closed and open triangles, respectively.

(C) Sketch of the setup to measure the amount of steady-state ATP-monomers as a function of CP concentration using formin-bound barbed ends. Tracer filaments are nucleated by exposing anchored formins to profilin and labeled actin (Alexa 488). These tracers are then exposed to steady-state solution containing unlabeled actin. The labeled segment of the filament appears to move away as unlabeled monomers are added by the formin at point of anchoring. Example kymographs show fast/slow elongation as indicators of high/low values of the steady-state amount of polymerizable actin monomers ($[PA]_{SS}+[A]_{SS}$).

(D) Representative kymographs of barbed ends elongating from anchored formins (yellow dotted line). F-actin in the flow was unlabeled, letting the labeled pointed-end region of tracer filaments move away as insertional polymerization occurs at formin-bound barbed end.

(E) Rate of formin elongation versus CP concentration (black closed circles) and corresponding steady-state concentrations of profilin-actin $[PA]_{SS}$ (red closed circles).

(F) Representative kymographs (two for each condition) of tracer barbed ends growing in the presence of CP and ADF.

(G) Diagram representing the synergy between CP and ADF in enhancing steady-state growth of individual filaments. Error bars represent SD. Each data point was averaged from at least 50 filaments over three experiments ($n > 50$).

All elongation rates in (B) (except at $\gamma = 0.75$) are statistically significant ($p < 0.05$; two-sample t test) compared to the rate at $\gamma = 0.5$. All elongation rates in (E) (except at $\gamma = 0.5$ and 0.75) are statistically significant ($p < 0.05$; two-sample t test) compared to the rate at $\gamma = 0.33$. All elongation rates in (G) were found to be statistically significant compared ($p < 0.05$; two-sample t test) to control (0 ADF and 0 CP).

monomer concentration seen here, thus ensuring that both F-actin and G-actin are present at steady state. Profilin concentration (3 μM) was kept high enough to ensure that most monomeric actin gets converted to profilin-actin and low enough that profilin does not compete with CP at barbed ends [48].

ADF establishes a higher ATP-monomer concentration and results in faster (but equal) global rates of barbed-end growth and pointed-end depolymerization (Figure 4C). This effect cumulates with CP-mediated funneling (Figures 4B and 4E) in a synergistic fashion (Figures 4D and 4E). The individual and cooperative regulations by ADF and CP have been demonstrated here at the single-filament scale, thanks to an original approach for direct TIRF-based measurement of monomer concentration. The growth rate measurements are consistent with the ones expected from a graphical representation of the contribution of barbed and pointed ends of filaments to the steady state of actin assembly (Figure 4E). Elongation rates of 24 su/s at 1μM ADF and 5 nM CP are in close agreement with estimates of lamellipodial barbed-end assembly rates (66 su/s) [49, 50]. Our results represent a major step forward in our understanding of the molecular mechanisms by which cells might control a steady-state monomer pool that

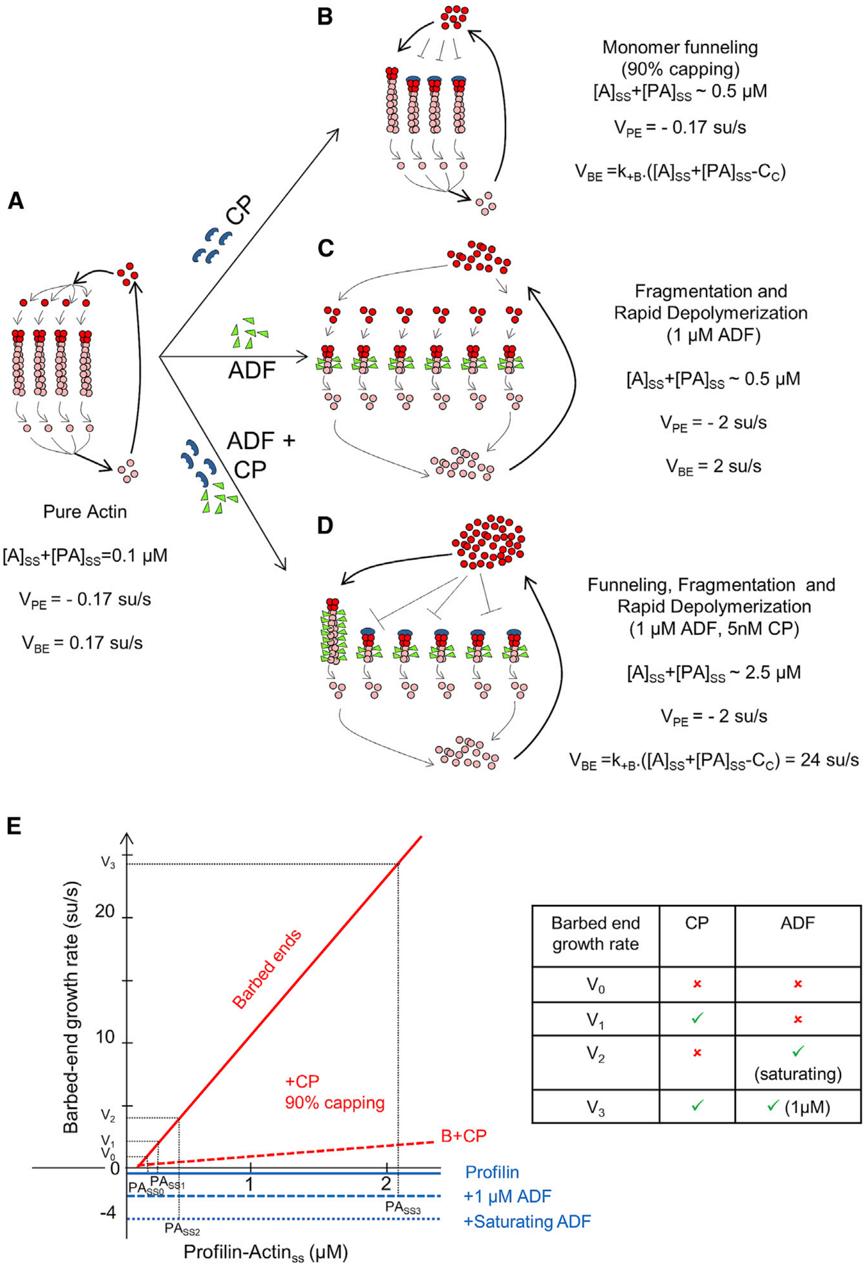

Barbed end growth rate	CP	ADF
V_0	×	×
V_1	✓	×
V_2	×	✓ (saturating)
V_3	✓	✓ (1μM)

Figure 4. Combined Effects of ADF and CP on Barbed-End Growth Rate at Steady State

(A) Control filaments (four shown) treadmill very slowly due to low steady-state polymerizable monomer (~0.1 μM; mainly profilin-actin), at which barbed-end growth rate ($V_{BE} = k_{+B}.([A]_{SS}+[PA]_{SS} - C_c)$; see Equations 5 and 6; STAR Methods) balances slow pointed-end depolymerization (V_{PE}~−0.17 su/s).

(B) In the presence of CP (90% capping), all pointed ends feed a fewer number (10%) of uncapped barbed ends, which individually grow faster.

(C) In the presence of ADF, enhanced pointed-end depolymerization (2 su/s at saturation by ADF) establishes a higher concentration $[PA_{SS}]$ (0.5 μM).

(D) In the presence of both ADF and capping protein (CP), $[PA_{SS}]$ further increases and non-capped barbed ends grow faster.

(E) Graphical representation of the effects of ADF and CP on the contribution of barbed- and pointed-end dynamics in establishing the steady state of actin assembly. Red and blue lines represent the change in elongation rates at filament barbed and pointed ends, respectively, versus monomeric actin concentration in the presence of 3 μM profilin. Due to the presence of profilin, pointed ends only depolymerize at constant rate: 0.17 su/s in absence of ADF (continuous line); 2 su/s in presence of 1 μM ADF (dashed line); and 5 su/s in presence of saturating ADF (dotted line). Barbed ends grow with 100% free (non-capped) barbed ends (continuous red line) or 90% capped barbed ends (dotted red line indicates bulk growth rate). The steady-state monomer concentration is first determined as the monomer concentration at which pointed end disassembly equals barbed end assembly bulk rate. The elongation rate V_i of individual barbed ends at steady state is then read on the graph as the rate of non-capped barbed end (continuous red line) at the steady-state monomer concentration. Conditions shown as examples are as follows: no regulator (V_0); + 2 nM CP: (V_1); + saturating ADF (V_2); and + both 1 μM ADF and CP (V_3).

determines the rate of cell migration. Our novel method can in the future be easily adapted to explore contributions of other effector proteins (e.g., Aip1, twinfilin, and coronin) to steady-state monomer pool and promises to be seminal, for instance in offering a quantitative measurement, at the single filament level, of the actual concentration of profilin-actin in various cell extracts, which is a major unsolved issue [1, 48, 51].

STAR★METHODS

Detailed methods are provided in the online version of this paper and include the following:

- KEY RESOURCES TABLE
- CONTACT FOR REAGENT AND RESOURCE SHARING
- EXPERIMENTAL MODEL AND SUBJECT DETAILS
- METHOD DETAILS
  - Protein purification
  - Purification of Profilin
  - Purification of Capping Protein
  - Purification of ADF
  - Purification and biotinylation of Formin mDia1
  - Single filament microscopy
  - Microscopy data acquisition and analysis
  - Determination of steady-state monomer concentration from barbed-end growth rate (Figures 2B, 3B, and 3E)
  - Calculation of increase in elongation rate as a function of CP concentration (Figures 3B and 3E)

- QUANTIFICATION AND STATISTICAL ANALYSIS
- DATA AND SOFTWARE AVAILABILITY

SUPPLEMENTAL INFORMATION

Supplemental Information includes two figures and five movies and can be found with this article online at http://dx.doi.org/10.1016/j.cub.2017.05.036.

AUTHOR CONTRIBUTIONS

S.S. and M.-F.C. conceived and designed the experiments, S.S. conducted the experiments and analyzed the data, and S.S. and M.-F.C. wrote the manuscript.

ACKNOWLEDGMENTS

We thank Bruce Goode, Jané Kondev, Lishibanya Mohapatra, Adam Johnston, Silvia Jansen, and Greg Hoeprich for stimulating discussions on actin disassembly and for critical feedback on the manuscript. We thank Julien Pernier for assistance in expression and purification of ADF and Bérengère Guichard for technical assistance in expression and purification of all other proteins. The work was supported by ERC advanced grant ‘‘Forcefulactin’’ no. 249982 and EC FP7 grant no. 241548 to M.-F.C.

Received: February 16, 2017
Revised: April 12, 2017
Accepted: May 9, 2017
Published: June 15, 2017

REFERENCES

1. Carlier, M.F., and Shekhar, S. (2017). Global treadmilling coordinates actin turnover and controls the size of actin networks. Nat. Rev. Mol. Cell Biol. Published online March 1, 2017. http://dx.doi.org/10.1038/nrm.2016.172.
2. Suarez, C., and Kovar, D.R. (2016). Internetwork competition for monomers governs actin cytoskeleton organization. Nat. Rev. Mol. Cell Biol. *17*, 799–810.
3. Shekhar, S., Pernier, J., and Carlier, M.F. (2016). Regulators of actin filament barbed ends at a glance. J. Cell Sci. *129*, 1085–1091.
4. Kiuchi, T., Ohashi, K., Kurita, S., and Mizuno, K. (2007). Cofilin promotes stimulus-induced lamellipodium formation by generating an abundant supply of actin monomers. J. Cell Biol. *177*, 465–476.
5. Aizawa, H., Sutoh, K., and Yahara, I. (1996). Overexpression of cofilin stimulates bundling of actin filaments, membrane ruffling, and cell movement in Dictyostelium. J. Cell Biol. *132*, 335–344.
6. Lappalainen, P., and Drubin, D.G. (1997). Cofilin promotes rapid actin filament turnover in vivo. Nature *388*, 78–82.
7. Rosenblatt, J., Agnew, B.J., Abe, H., Bamburg, J.R., and Mitchison, T.J. (1997). Xenopus actin depolymerizing factor/cofilin (XAC) is responsible for the turnover of actin filaments in Listeria monocytogenes tails. J. Cell Biol. *136*, 1323–1332.
8. Meberg, P.J., and Bamburg, J.R. (2000). Increase in neurite outgrowth mediated by overexpression of actin depolymerizing factor. J. Neurosci. *20*, 2459–2469.
9. Kanellos, G., and Frame, M.C. (2016). Cellular functions of the ADF/cofilin family at a glance. J. Cell Sci. *129*, 3211–3218.
10. Ono, S. (2007). Mechanism of depolymerization and severing of actin filaments and its significance in cytoskeletal dynamics. Int. Rev. Cytol. *258*, 1–82.
11. Wang, R., and Carlsson, A.E. (2015). How capping protein enhances actin filament growth and nucleation on biomimetic beads. Phys. Biol. *12*, 066008.
12. Akin, O., and Mullins, R.D. (2008). Capping protein increases the rate of actin-based motility by promoting filament nucleation by the Arp2/3 complex. Cell *133*, 841–851.
13. Andrianantoandro, E., and Pollard, T.D. (2006). Mechanism of actin filament turnover by severing and nucleation at different concentrations of ADF/cofilin. Mol. Cell *24*, 13–23.
14. Michelot, A., Berro, J., Guérin, C., Boujemaa-Paterski, R., Staiger, C.J., Martiel, J.L., and Blanchoin, L. (2007). Actin-filament stochastic dynamics mediated by ADF/cofilin. Curr. Biol. *17*, 825–833.
15. Carlier, M.F., Laurent, V., Santolini, J., Melki, R., Didry, D., Xia, G.X., Hong, Y., Chua, N.H., and Pantaloni, D. (1997). Actin depolymerizing factor (ADF/cofilin) enhances the rate of filament turnover: implication in actin-based motility. J. Cell Biol. *136*, 1307–1322.
16. Carlier, M.F., and Pantaloni, D. (1997). Control of actin dynamics in cell motility. J. Mol. Biol. *269*, 459–467.
17. Pollard, T.D. (1986). Rate constants for the reactions of ATP- and ADP-actin with the ends of actin filaments. J. Cell Biol. *103*, 2747–2754.
18. Watanabe, N., and Mitchison, T.J. (2002). Single-molecule speckle analysis of actin filament turnover in lamellipodia. Science *295*, 1083–1086.
19. Loisel, T.P., Boujemaa, R., Pantaloni, D., and Carlier, M.F. (1999). Reconstitution of actin-based motility of Listeria and Shigella using pure proteins. Nature *401*, 613–616.
20. Pantaloni, D., Le Clainche, C., and Carlier, M.F. (2001). Mechanism of actin-based motility. Science *292*, 1502–1506.
21. Pollard, T.D., and Borisy, G.G. (2003). Cellular motility driven by assembly and disassembly of actin filaments. Cell *112*, 453–465.
22. Chin, S.M., Jansen, S., and Goode, B.L. (2016). TIRF microscopy analysis of human Cof1, Cof2, and ADF effects on actin filament severing and turnover. J. Mol. Biol. *428*, 1604–1616.
23. Suarez, C., Roland, J., Boujemaa-Paterski, R., Kang, H., McCullough, B.R., Reymann, A.C., Guérin, C., Martiel, J.L., De la Cruz, E.M., and Blanchoin, L. (2011). Cofilin tunes the nucleotide state of actin filaments and severs at bare and decorated segment boundaries. Curr. Biol. *21*, 862–868.
24. Didry, D., Carlier, M.F., and Pantaloni, D. (1998). Synergy between actin depolymerizing factor/cofilin and profilin in increasing actin filament turnover. J. Biol. Chem. *273*, 25602–25611.
25. Ressad, F., Didry, D., Xia, G.X., Hong, Y., Chua, N.H., Pantaloni, D., and Carlier, M.F. (1998). Kinetic analysis of the interaction of actin-depolymerizing factor (ADF)/cofilin with G- and F-actins. Comparison of plant and human ADFs and effect of phosphorylation. J. Biol. Chem. *273*, 20894–20902.
26. Hug, C., Jay, P.Y., Reddy, I., McNally, J.G., Bridgman, P.C., Elson, E.L., and Cooper, J.A. (1995). Capping protein levels influence actin assembly and cell motility in dictyostelium. Cell *81*, 591–600.
27. Wiesner, S., Helfer, E., Didry, D., Ducouret, G., Lafuma, F., Carlier, M.F., and Pantaloni, D. (2003). A biomimetic motility assay provides insight into the mechanism of actin-based motility. J. Cell Biol. *160*, 387–398.
28. Edwards, M., Zwolak, A., Schafer, D.A., Sept, D., Dominguez, R., and Cooper, J.A. (2014). Capping protein regulators fine-tune actin assembly dynamics. Nat. Rev. Mol. Cell Biol. *15*, 677–689.
29. Schafer, D.A., Jennings, P.B., and Cooper, J.A. (1996). Dynamics of capping protein and actin assembly in vitro: uncapping barbed ends by polyphosphoinositides. J. Cell Biol. *135*, 169–179.
30. Nadkarni, A.V., and Brieher, W.M. (2014). Aip1 destabilizes cofilin-saturated actin filaments by severing and accelerating monomer dissociation from ends. Curr. Biol. *24*, 2749–2757.
31. Jansen, S., Collins, A., Chin, S.M., Ydenberg, C.A., Gelles, J., and Goode, B.L. (2015). Single-molecule imaging of a three-component ordered actin disassembly mechanism. Nat. Commun. *6*, 7202.
32. Niedermayer, T., Jégou, A., Chièze, L., Guichard, B., Helfer, E., Romet-Lemonne, G., Carlier, M.F., and Lipowsky, R. (2012). Intermittent depolymerization of actin filaments is caused by photo-induced dimerization of actin protomers. Proc. Natl. Acad. Sci. USA *109*, 10769–10774.
33. Shekhar, S., and Carlier, M.F. (2016). Single-filament kinetic studies provide novel insights into regulation of actin-based motility. Mol. Biol. Cell *27*, 1–6.

34. Jégou, A., Niedermayer, T., Orbán, J., Didry, D., Lipowsky, R., Carlier, M.F., and Romet-Lemonne, G. (2011). Individual actin filaments in a microfluidic flow reveal the mechanism of ATP hydrolysis and give insight into the properties of profilin. PLoS Biol. *9*, e1001161.

35. Kuhn, J.R., and Pollard, T.D. (2005). Real-time measurements of actin filament polymerization by total internal reflection fluorescence microscopy. Biophys. J. *88*, 1387–1402.

36. Jiao, Y., Walker, M., Trinick, J., Pernier, J., Montaville, P., and Carlier, M.F. (2014). Mutagenetic and electron microscopy analysis of actin filament severing by Cordon-Bleu, a WH2 domain protein. Cytoskeleton *71*, 170–183.

37. Johnston, A.B., Collins, A., and Goode, B.L. (2015). High-speed depolymerization at actin filament ends jointly catalysed by twinfilin and Srv2/CAP. Nat. Cell Biol. *17*, 1504–1511.

38. Galkin, V.E., Orlova, A., Schröder, G.F., and Egelman, E.H. (2010). Structural polymorphism in F-actin. Nat. Struct. Mol. Biol. *17*, 1318–1323.

39. McCullough, B.R., Grintsevich, E.E., Chen, C.K., Kang, H., Hutchison, A.L., Henn, A., Cao, W., Suarez, C., Martiel, J.L., Blanchoin, L., et al. (2011). Cofilin-linked changes in actin filament flexibility promote severing. Biophys. J. *101*, 151–159.

40. Bamburg, J.R., McGough, A., and Ono, S. (1999). Putting a new twist on actin: ADF/cofilins modulate actin dynamics. Trends Cell Biol. *9*, 364–370.

41. Vartiainen, M.K., Mustonen, T., Mattila, P.K., Ojala, P.J., Thesleff, I., Partanen, J., and Lappalainen, P. (2002). The three mouse actin-depolymerizing factor/cofilins evolved to fulfill cell-type-specific requirements for actin dynamics. Mol. Biol. Cell *13*, 183–194.

42. Jégou, A., Carlier, M.F., and Romet-Lemonne, G. (2013). Formin mDia1 senses and generates mechanical forces on actin filaments. Nat. Commun. *4*, 1883.

43. Gressin, L., Guillotin, A., Guérin, C., Blanchoin, L., and Michelot, A. (2015). Architecture dependence of actin filament network disassembly. Curr. Biol. *25*, 1437–1447.

44. Pfaendtner, J., De La Cruz, E.M., and Voth, G.A. (2010). Actin filament remodeling by actin depolymerization factor/cofilin. Proc. Natl. Acad. Sci. USA *107*, 7299–7304.

45. Walsh, T.P., Weber, A., Higgins, J., Bonder, E.M., and Mooseker, M.S. (1984). Effect of villin on the kinetics of actin polymerization. Biochemistry *23*, 2613–2621.

46. Bombardier, J.P., Eskin, J.A., Jaiswal, R., Corrêa, I.R., Jr., Xu, M.Q., Goode, B.L., and Gelles, J. (2015). Single-molecule visualization of a formin-capping protein 'decision complex' at the actin filament barbed end. Nat. Commun. *6*, 8707.

47. Shekhar, S., Kerleau, M., Kühn, S., Pernier, J., Romet-Lemonne, G., Jégou, A., and Carlier, M.F. (2015). Formin and capping protein together embrace the actin filament in a ménage à trois. Nat. Commun. *6*, 8730.

48. Pernier, J., Shekhar, S., Jegou, A., Guichard, B., and Carlier, M.F. (2016). Profilin interaction with actin filament barbed end controls dynamic instability, capping, branching, and motility. Dev. Cell *36*, 201–214.

49. Miyoshi, T., Tsuji, T., Higashida, C., Hertzog, M., Fujita, A., Narumiya, S., Scita, G., and Watanabe, N. (2006). Actin turnover-dependent fast dissociation of capping protein in the dendritic nucleation actin network: evidence of frequent filament severing. J. Cell Biol. *175*, 947–955.

50. Miyoshi, T., and Watanabe, N. (2013). Can filament treadmilling alone account for the F-actin turnover in lamellipodia? Cytoskeleton *70*, 179–190.

51. Rotty, J.D., Wu, C., Haynes, E.M., Suarez, C., Winkelman, J.D., Johnson, H.E., Haugh, J.M., Kovar, D.R., and Bear, J.E. (2015). Profilin-1 serves as a gatekeeper for actin assembly by Arp2/3-dependent and -independent pathways. Dev. Cell *32*, 54–67.

52. Carlier, M.F., Romet-Lemonne, G., and Jégou, A. (2014). Actin filament dynamics using microfluidics. Methods Enzymol. *540*, 3–17.

53. Pollard, T.D., and Cooper, J.A. (1986). Actin and actin-binding proteins. A critical evaluation of mechanisms and functions. Annu. Rev. Biochem. *55*, 987–1035.

54. Kinosian, H.J., Selden, L.A., Gershman, L.C., and Estes, J.E. (2002). Actin filament barbed end elongation with nonmuscle MgATP-actin and MgADP-actin in the presence of profilin. Biochemistry *41*, 6734–6743.

STAR★METHODS

KEY RESOURCES TABLE

REAGENT or RESOURCE	SOURCE	IDENTIFIER
Bacterial and Virus Strains		
BL21 (DE3)	TransGen Biotech	Cat#CD601-02
Chemicals, Peptides, and Recombinant Proteins		
Magnesium Chloride ($MgCl_2$)	Sigma-Aldrich	Cat#M8266
IPTG	AMRESCO	Cat#0487
Protease inhibitor cocktail	Sigma-Aldrich	Cat#P8340
Potassium Chloride (KCl)	Sigma-Aldrich	Cat#P9541
Calcium Chloride ($CaCl_2$)	Sigma-Aldrich	Cat#C4901
Phenylmethylsulfonyl fluoride (PMSF)	Sigma-Aldrich	Cat#10837091001
Adenosine 5′-triphosphate disodium salt hydrate (ATP)	Sigma-Aldrich	Cat#A2383
1,4-Dithiothreitol (DTT)	Sigma-Aldrich	Cat#10197777001
Ethylenediaminetetraacetic acid (EDTA)	Sigma-Aldrich	Cat#E9884
Ethylene glycol-bis(2-aminoethylether)-N,N,N′, N′-tetraacetic acid (EGTA)	Sigma-Aldrich	Cat#E3889
Sodium Chloride	Sigma-Aldrich	Cat#S3014
Imidazole	Sigma-Aldrich	Cat#I5513
Tris	Sigma-Aldrich	Cat#10708976001
PIPES	Sigma-Aldrich	Cat#P1851
Urea	Sigma-Aldrich	Cat#U5378
Tween	Sigma-Aldrich	Cat#P1379
SNAP-Biotin	New England Biolabs	Cat#S9110S
PLL(20)-g[3.5]- PEG(2)	SuSos A.G.	N/A
BSA	Sigma-Aldrich	Cat#A2153
Muscle acetone powder from rabbit	Sigma-Aldrich	M6890
Alexa Fluor 488 NHS Ester	Thermo Fisher Scientific	Cat#A20000
Critical Commercial Assays		
Sylgard 184 silicone elastomer kit	Dow Corning Corporation	Cat#1064291
Software and Algorithms		
ImageJ	http://rsb.info.nih.gov/ij/	N/A
MetaMorph	Molecular Devices	N/A
Other		
Inverted TIRF microscope	Olympus Corporation	IX71
60 ×, 1.4 NA oil-immersion objective	Olympus Corporation	N/A
Vivaspin 6	Sartorius A.G.	Cat#VS0601
Vivaspin 20	Sartorius A.G.	Cat#VS2002
ÄKTA FPLC system	GE Healthcare Life Sciences	Cat#18190026
HisTrap FF Crude 5ml Column	GE Healthcare Life Sciences	Cat#17-5286-01
HiLoad 16/60 Superdex 200 prep grade Column	GE Healthcare Life Sciences	Cat#28989335
ReSource Q column	GE Healthcare Life Sciences	Cat#17-1179-01
ReSource S column	GE Healthcare Life Sciences	Cat#17-1180-01
Poly-L-Proline Column	Sigma Aldrich	Cat# P2129

CONTACT FOR REAGENT AND RESOURCE SHARING

Further information and requests for resources and reagents should be directed to and will be fulfilled by the Lead Contact, Shashank Shekhar (shashank.shekhar@yahoo.com).

EXPERIMENTAL MODEL AND SUBJECT DETAILS

No cell or animal experiments were conducted.

METHOD DETAILS

Protein purification

Purification and fluorescent labelling of Actin

Acetone powder of rabbit muscle extract (9 g) was mixed in 270 mL of extraction X buffer (2 mM Tris-HCl pH 7.8, 0.5 mM ATP, 0.1 mM $CaCl_2$, 1 mM DTT, 0.01% NaN_3) in a 500 mL beaker kept on ice using a glass stirring rod for a total of 40 min. The solution was then centrifuged at 25,000 x g for 45 min at 4°C and the supernatant filtered over glass wool placed in a funnel. To the filtered volume, solid potassium chloride (KCl) was added to have a final concentration of 3.3 M. The beaker was then placed in a warm water bath at 20-25°C and mixing was continued by magnetic stirring until temperature reached 15°C. The solution was then placed on ice without any further mixing until temperature reached 5°C. The solution was once again centrifuged at 25,000 x g for 30 min at 4°C. The pellet, which consisted of alpha-actinin-actin gel, was discarded and the supernatant was filtered over glass wool. The filtrate was then dialyzed overnight at 4°C against 32 volumes of dialysis buffer D1 (2 mM Tris-HCl, pH 7.8, 1 mM $MgCl_2$, 1 mM DTT). KCl was added to the dialyzate to a final concentration of 0.8 M and mixing continued with magnetic agitation for 1.5 hr at 4°C followed by centrifugation for 3.5 hr at 100,000 x g at 4°C. The supernatant containing tropomyosin was discarded and the pellet of F-actin was gently resuspended in a total volume of 25 ml of extraction X buffer. 75 μl of 1 M $MgCl_2$ and 375 μl of 4 M KCl was added and the volume was completed to 38.6 mL with extraction X-buffer and homogenized. A second round of elimination of tropomyosin was performed by adding 9 mL of 4M KCl, adjusting the volume to 50 mL with X-buffer. The solution was then mixed with magnetic agitation for 1.5 hr at 4°C and centrifuged for 3.5 hr at 100,000 x g at 4°C. The tropomyosin-enriched supernatant was discarded and the pellet of F-actin was resuspended in 30 mL of extraction X-buffer (4°C), homogenized with a potter on ice and dialyzed against 2 l of G-buffer for 48 hr at 4°C. The dialysis buffer was then changed and the dialysis continued overnight against 1 l of fresh G-Buffer. Using a tip sonicator, the solution was sonicated three times with 30 s pulses at low power by dipping the tip in the dialysis bag and the dialysis was continued overnight in fresh 1 l G-buffer. The solution was centrifuged for 1.5 hr at 400,000 x g at 4°C and the supernatant was loaded on to a pre-equilibrated 2.5 × 100 cm Superdex 300 size exclusion column to separate actin oligomers from G-actin. The last two thirds of the main monomer peak were collected, their absorbance at 290 nm was measured and concentration was calculated (molar extinction coefficient of actin at 290 nm = 0.617 mg/ml and the molecular weight is 42 kDa). The typical yield of this preparation is 40 mL at 55 ± 10 μM (2.3 ± 0.4 mg/ml) G-actin. Purified G-actin can be stored on ice for up to 3 weeks.

Purified actin was labeled by reacting Alexa 488 NHS Ester dye with F-actin exposed surface lysine residues as follows. 2 mL of purified G-actin (concentration ~50μM) was polymerized by dialyzing it overnight at 4°C against modified F-buffer (20 mM PIPES pH 6.9, 0.2 mM $CaCl_2$, 0.2 mM ATP and 100 mM KCl). The F-actin was labeled by incubation with the dye (final concentration 0.25 mM) for two hours at room temperature in the dark on a rotating wheel. Labeled F-actin was then centrifuged at 450,000 x g for 30 min at room temperature. The pellet of labeled F-actin was resuspended in 2 mL of G-buffer (5 mM Tris-HCl pH 7.8, 0.1 mM $CaCl_2$, 0.2 M ATP, 0.01% NaN_3 and 5 mM DTT) homogenized using a potter and then allowed to depolymerize on ice in the dark for 2 hr. This solution was re-polymerized by adding $MgCl_2$ and KCl (final concentrations of 1 mM and 100 mM) and incubating on ice for one hour followed by centrifugation at 450,000 x g for 30 min at 4°C. The supernatant was discarded and the pellet of polymerizable labeled F-actin was homogenized in 2 mL of G-buffer using a potter. This solution was dialyzed at 4°C in the dark overnight against 1 l of G-buffer and was once again centrifuged 450,000 x g for 30 min at 4°C next morning. The supernatant contains labeled monomeric actin. The concentration and labeling efficiency of G-actin was then determined by measuring the absorbance at 280 nm (A280) and at 495 nM (A495). Calculate the concentration of actin and dye:

$$[Actin] = \frac{(A_{280} - A_{494} * CF_{280})}{\varepsilon_{280}}$$

$$[Dye] = \frac{(A_{494})}{\varepsilon_{D494}}$$

$$\%\ \text{labelling} = \frac{[Dye]}{[Actin]} * 100.$$

Molar extinction coefficient for actin, $\varepsilon_{280} = 45{,}840\ M^{-1}cm^{-1}$ and for Alexa 488 fluorophore, $\varepsilon_{D494} = 71{,}000\ M^{-1}cm^{-1}$ and the correction factor Alexa 488 fluorophore at 280 nm, $CF_{280} = 0.11$.

Purification of Profilin

E. coli BL21 DE3 cells were transformed with a plasmid containing human Profilin. The transformed cells were then grown in LB in presence of selection antibiotics. Protein expression was then induced by incubating overnight with 1 mM IPTG. The cells were

washed with PBS next morning and resuspended in lysis buffer (50 mM Tris-HCl pH 7.3, 5 mM EGTA, 0.1 mM EDTA, 50 mM KCl, 8 M Urea, 0.1% Tween-20, 10 mM DTT, 1 mM PMSF, Protease Inhibitors). The solution was incubated for 30 min with rotation at 4°C followed by sonication with a tip sonicator (8*30 s pulses) while keeping the sample on ice. The lysate was then ultracentrifuged for 185,000 x g for 30 min at 4°C and the supernatant was dialyzed overnight at 4°C against 2.2 l of dialysis buffer (50 mM Tris-HCl pH 7.3, 1 mM EGTA, 0.1 mM EDTA, 50 mM KCl, 1 mM DTT). A dialysis membrane with a cut-off 12-14,000 Da was used. Prepare Elution Buffer (50 mM Tris-HCl pH 7.3, 5 mM EGTA, 0.1 mM EDTA, 50 mM KCl, 8 M Urea, 10 mM DTT) and washing buffer (3 Volumes of dialysis buffer + 1 volume of elution buffer).

Next morning, a Poly-L-Proline column connected to an FPLC system was equilibrated with 4 column volumes of Washing Buffer and the dialyzed lysate was flowed through the column. The profilin-bound column was first washed with washing buffer and the protein was then eluted out by flowing through elution buffer. Eluted protein was further dialyzed overnight against dialysis buffer (dialysis membrane cut-off 12-14,000 Da). Dialyzed protein was concentrated with Vivaspin 20 (10,000 Da) and once again dialyzed overnight against conservation buffer (10 mM Tris-HCl pH 7.5, 50 mM KCl, 1 mM DTT). Protein concentration was determined by measuring the absorbance at 280 nm (Absorbance of a 0.1% solution = 1.15 and Profilin molecular weight = 14,800 Da). Purified protein was flash frozen in liquid nitrogen and stored at −80°C.

Purification of Capping Protein

E. coli BL21 DE3 cells were transformed with a plasmid containing mouse His-$\alpha1\beta1$ Capping Protein. The transformed cells were then grown in LB in presence of selection antibiotics. Protein expression was then induced by incubating overnight with 1 mM IPTG. The cells were washed with PBS and resuspended in lysis buffer (20 mM $NaPO_4$ pH 7.8, 500 mM NaCl, 1 mM DTT, 15 mM Imidazole, 0.1 mM EDTA, 0.5%Tween, 0.1 mM PMSF + Protease Inhibitors). The solution was mixed with magnetic agitation for 20 min at 4°C followed by addition of lysozyme (final concentration ~1mg/ml). The solution was further incubated for 15 min on ice and sonicated with a tip sonicator keeping the tubes on ice. The lysate was then ultracentrifuged at 150,000 x g for 50 min at 4°C. The supernatant was then flowed through a HisTrap column connected to a Fast Protein Liquid Chromatography (FPLC) system and the column was then washed with the washing buffer (20 mM $NaPO_4$ pH 7.8, 500 mM NaCl, 1 mM DTT, 15 mM Imidazole) to remove proteins non-specifically bound to the column. Capping protein was then eluted by flowing through elution buffer (20 mM $NaPO_4$ pH 7.8, 500 mM NaCl, 1 mM DTT and 500 mM Imidazole) over the column. The eluted protein was then concentrated with Vivaspin 6 tubes (30 kDa) up to a final volume of about 2 mL and then loaded on a pre-equilibrated gel filtration column (HiLoad 16/60 Superdex 200 prep grade) connected to an FPLC system (Gel filtration buffer: 20 mM Tris pH 7.5, 50 mM KCl, 1 mM DTT). Fractions containing the protein were combined and protein concentration was determined by measuring the absorbance at 280 nm (Molar extinction coefficient at 280 nm = 76,300 $M^{-1}cm^{-1}$.). Purified protein was flash frozen in liquid nitrogen and stored at −80°C.

Purification of ADF

E. coli BL21 DE3 cells were transformed with a plasmid containing human ADF. The transformed cells were then grown in LB in presence of selection antibiotics. The cells were then resuspended in 100 mL of Lysis Buffer (10 mM Tris-HCl pH 7.8, 1 mM EDTA, 1% Triton 100X, 5% Glycerol, 1 mM DTT and Protease Inhibitors) to which 0.5 mg/ml of lysozyme was added and the mix was incubated for 30 min with rotation at 4°C. The incubation was continued for another 30 min after addition of 10 μg/ml of DNase I. 5 mM MgCl2 and 0.1 mM PMSF was then added to the solution followed by sonication by a tip sonicator to completely lyse the cells. The solution was kept on ice during sonication. The lysate was then centrifuged for 30 min at 18,000 x g at 4°C and the supernatant was dialyzed (cut-off 12-14000 Da) overnight at 4°C against 2 l of Buffer QA (10 mM Tris-HCl pH 7.5, 50 mM NaCl, 5 mM DTT, 0.2 mM EGTA, 0.1% NaN_3). The dialyzed lysate was loaded on a ReSource Q ion-exchange column connected to an FPLC system and sequentially washed with 5 column volumes of water, Buffer QA, Buffer QB (10 mM Tris-HCl pH 7.5, 1 M NaCl, 5 mM DTT, 0.2 mM EGTA, 0.1% NaN_3) and Buffer QA. The column was then washed with Buffer QA. The flowthrough was conserved and concentrated using a Vivaspin 20 (10 kDa). Concentrated protein was dialyzed against 1 l Buffer SA (10 mM PIPES pH 6.5, 25 mM NaCl, 5 mM DTT, 0.2 mM EGTA, 0.01% NaN_3). Next morning, the dialyzed protein was loaded on a ReSource S ion-exchange column that had been washed with 5 column volumes of water, Buffer SA, Buffer SB (10 mM PIPES pH 6.5, 50 mM NaCl, 5 mM DTT, 0.2 mM EGTA, 0.01% NaN_3) and Buffer SA. The bound protein was then eluted from the column with buffer SB and the fractions containing the protein were combined and concentrated using Vivaspin 20 (10 kDa). The concentrated protein was then dialyzed overnight at 4°C against 1 l of conservation buffer (5 mM Tris-HCl pH 7.5, 0.01% NaN_3, 1 mM DTT) (dialysis membrane cut-off 12-14 kDa) followed by ultracentrifugation at 4°C at 450,000 x g next morning. Protein concentration was determined by measuring the absorbance at 280 nm (Absorbance of a 0.1% solution = 0.645, and ADF molecular weight = 16,000 Da). Purified protein was flash frozen in liquid nitrogen and stored at −80°C.

Purification and biotinylation of Formin mDia1

E. coli BL21 DE3 cells were transformed with formin mDia1 plasmid His-SNAP-FH1-FH2-DAD. The transformed cells were then grown in LB in presence of selection antibiotics. The cells were then induced with IPTG overnight and resuspended next morning in 100 mL of Lysis Buffer (20 mM $NaPO_4$ pH 7.8, 500 mM NaCl, 15 mM Imidazole, 0.1 mM EDTA, 0.5% Tween-20, 1 mM DTT, 1 mM PMSF, Protease Inhibitors) to which 0.5 mg/ml of lysozyme was added. The solution was incubated for 30 min with rotation at 4°C followed by sonication with a tip sonicator (8*30 s pulses) while keeping the sample on ice. The lysate was then ultracentrifuged for 185,000 x g for 30 min at 4°C and the supernatant was loaded on to an equilibrated HisTrap FF crude 5 mL column connected to a

FPLC system (equilibration buffer: 20 mM $NaPO_4$ pH 7.8, 500 mM NaCl, 15 mM Imidazole, 1 mM DTT). The column was once again washed with the equilibration buffer to remove non-specifically bound protein. The bound protein was then eluted by flowing elution buffer through the column (20 mM $NaPO_4$ pH 7.8, 500 mM NaCl, 15 mM Imidazole, 1 mM DTT). The eluted protein was then dialyzed overnight (membrane cutoff 12-14000 Da) against the gel filtration buffer (20 mM Tris-HCl pH 7.5, 50 mM KCl, 1 mM DTT). The protein was then loaded onto HiLoad 16/60 Superdex 200 prep grade Column connected to a FPLC system and fractions were collected and their absorbance at 280 nm was measured. Fractions containing the protein were pooled together and protein concentration was determined by measuring the absorbance at 280 nm (calculated molar extinction coefficient = 42400 $M^{-1}cm^{-1}$). Purified SNAP-mDia1 was then biotinylated by reacting benzylguanine–biotin (New England Biolabs) following vendor prescribed procedure. Biotinylated protein was flash frozen with liquid nitrogen and store at $-80°C$.

Single filament microscopy

The kinetics of individual filament assembly/disassembly was monitored using microfluidics coupled to fluorescence microscopy [47] ([52] for review). A 40 μm high PDMS mold with 3 inlets was stuck onto a clean coverslip. First, the coverslip surface was functionalized and passivated using a 1:40 PLL-PEG-Biotin:PLL-PEG mixture at 1 mg/mL in PBS, for 30 min at room temperature. The chamber was rinsed and further incubated with 10 μg/mL neutravidin in PBS for 5 min. Filaments were then initiated either from biotinylated spectrin-actin seeds exposing their barbed ends in a distal position, or Gelsolin-actin seeds or from immobilized biotinylated SNAP-mDia1 (FH1-FH2-DAD) formin with their pointed ends in a distal position, thus allowing insertional filament assembly (incubation concentration for each ligand is 100 pM) [47]. This gives about 25-50 filaments per field of view. Gelsolin-actin GA2 seeds were prepared by mixing 2.5 molar equivalent of G-actin to Gelsolin in G-buffer. Standard conditions for growing tracer filaments from spectrin-actin seeds is 1 μM actin and 4 μM profilin and for nucleating filaments from anchored formins is 1 μM actin and 1 μM profilin. Actin was 10% Alexa 488 labeled in assays where filaments pointed ends were anchored by spectrin-actin seeds. When filaments were assembled from immobilized formin (anchored growing barbed ends), filaments were initiated with 10% Alexa 488 labeled actin, then elongated from unlabeled actin and their dynamics were monitored [47]. All experiments were carried out at room temperature in F-buffer (5 mM Tris-HCl pH 7.8, 2 mM ATP, 1 mM $MgCl_2$, 0.2 mM EGTA, 50 mM KCl, 10 mM DTT, and 1 mM DABCO). All filaments in the field of view were taken into account (n~20-30 per field of view). They were all analyzed and their rates of elongation/depolymerization measured. Each experiment was repeated at least 3 times and the filaments in the 3 fields of view were pooled together.

Microscopy data acquisition and analysis

Actin filaments were imaged on an Olympus IX71 TIRF microscope equipped with a 60x objective and a Cascade II EMCCD camera (Photometrics). Images were analyzed using the ImageJ Kymograph plugin and the slopes were measured to determine the elongation/depolymerization rate of individual filaments. One actin subunit contributes to 2.7 nm of the filament length. Background fluorescence was subtracted automatically using the built-in rolling ball background subtraction algorithm (rolling ball radius 5 pixels).

Determination of steady-state monomer concentration from barbed-end growth rate (Figures 2B, 3B, and 3E)

Microfluidics-assisted TIRFM experiments aiming at monitoring the dynamics of free barbed ends in the presence of CP and ADF were performed using a flow of F-actin assembled at steady state in the presence of 3 μM profilin. “Tracer filaments” with free barbed ends were first initiated on the coverslip from anchored spectrin-actin seeds. The tracer barbed ends were then exposed to a flow containing the steady-state solution. In the steady-state mix of F-actin and profilin (with and without CP or ADF), ATP-monomers predominantly consist of profilin-actin (in addition to a very small amount of G-actin i.e., ~nM, see relevant Equations 4, 5, and 6 below). Profilin at 3 μM ensures that these monomers cannot add to pointed ends, and that the medium is biochemically more similar to physiological conditions. When the steady-state mix containing F-actin and polymerizable monomers is flowed over the tracer barbed ends, the tracer filaments elongate at a rate V, which is proportional to the concentration of polymerizable monomers ($[A]_{SS}+[PA]_{SS}$) in the mix. The concentration of each of the two species of polymerizable monomers can then be calculated using the following equations:

Parameter definition:

1. V = rate of barbed end elongation at steady state (as measured).
2. $[A]_{ss} + [PA]_{SS}$ = total concentration of polymerizable monomers at steady state (to be determined).
3. $[P]_{total}$ = total concentration of profilin = 3 μM
4. C_C = critical concentration for assembly of either A or PA at the barbed end of a filament = 0.08 μM [53].
5. K_P = Equilibrium dissociation constant for profilin-actin complex = 0.1 μM [54]
6. k_+ = on-rate of polymerizable monomers (G-actin and Profilin-Actin) at the barbed end = 10 $s^{-1}\mu M^{-1}$ [53] [48]

$$V = k_+ . \left([A]_{SS} + [PA]_{SS} - C_C\right) \tag{1}$$

$$P_{total} = [P]_{SS} + [PA]_{SS} \tag{2}$$

$$K_P = \frac{[P]_{SS} \cdot [A]_{SS}}{[PA]_{SS}} \tag{3}$$

Combining the laws of mass action and mass conservation into Equation 1 leads to:

$$[A]_{SS}^2 + [A]_{SS} \cdot \left[K_P + P_{total} - C_C - \frac{V}{k_+}\right] - K_P \cdot \left[C_C + \frac{V}{k_+}\right] = 0 \tag{4}$$

from which the values of $[A]_{SS}$ and $[PA]_{SS}$ can be derived at each measured value of V, using the known values of parameters K_P, $[P]_{total}$ and C_C

$$[A]_{SS} = \frac{-\left(K_P + P_{total} - C_C - \frac{V}{k_+}\right) \pm \sqrt{\left(K_P + P_{total} - C_C - \frac{V}{k_+}\right)^2 + 4.K_P.\left(C_C + \frac{V}{k_+}\right)}}{2} \tag{5}$$

$$[PA]_{SS} = P_{total} \cdot \frac{[A]_{SS}}{[A]_{SS} + K_P} \tag{6}$$

Calculation of increase in elongation rate as a function of CP concentration (Figures 3B and 3E)

Pointed-ends of pure actin filaments disassemble at a constant rate of about 0.17 su/s in absence of ADF (Figure 1C). An F-actin mix at steady-state therefore contains very low amount of polymerizable monomers. If Capping Protein is added to the mix such that majority of (> 90%) of barbed ends in the mix are capped, the value of $[A]_{SS}$ and $[PA]_{SS}$ increase, and the elongation rate of tracer barbed ends increases accordingly.

At a given concentration of Capping Protein, [CP], the fraction of bulk actin filaments capped at a given time is given by:

$$\gamma = \frac{[CP]}{[CP] + K_C} \tag{7}$$

where K_C = 0.1nM [29] is the equilibrium dissociation constant for CP binding to barbed ends.

Specifically, if J^P_{SS} is the steady-state rate of pointed-end disassembly (0.17 su/s in absence of ADF), the elongation rate of few non-capped barbed ends (or tracer barbed ends) J^B_{free} is given by the equation below [16]:

$$J^B_{free} = \frac{-J^P_{SS}}{(1-\gamma)}. \tag{8}$$

This equation is used to calculate the expected elongation rate in presence of varying amount of CP in Figure 3B. Equation 5 and 6 were used to calculate the actual amount of polymerizable monomers from the measured elongation rate of tracer barbed ends. Note that profilin amplifies the effect of capping by establishing a higher steady-state amount of monomers than if only free G-actin contributed in barbed end growth (Figure 3B).

QUANTIFICATION AND STATISTICAL ANALYSIS

For single-filament imaging, a field of view containing at least 20-30 filaments was chosen and time-lapsed images were acquired for acquired for at least 30 frames (over at least 150 s). The elongation/depolymerization rates of all the filaments in the field of view were measured. The experiment was repeated 3 times and the results from the three independent experiments were cumulated (minimum number of filaments analyzed = 50). The average and standard deviation from the pooled data was determined. All error bars plotted in the figures represent the standard deviation of the pooled data. n, the exact number of filaments measured for each condition, is mentioned in respective figure legends.

For Figures 1C, 1D, 2B, 3B, 3E, and 3G the statistical significance was ascertained using two-sample t test using Origin data analysis software (OriginLab Corporation), and $p < 0.05$ was taken as the threshold for significance. In each graph, the lowest rate of elongation/depolymerization (seen for the leftmost data point on the x axis) is taken as the base value to which all the other rates are tested for statistical significance. Each figure legend contains the details for how statistical significance was tested.

DATA AND SOFTWARE AVAILABILITY

A custom written script in ImageJ was used for preparing kymographs for the entire field of view, this script is available on request from the Lead Contact.

Current Biology, Volume *27*

Supplemental Information

Enhanced Depolymerization of Actin Filaments by ADF/Cofilin and Monomer Funneling by Capping Protein Cooperate to Accelerate Barbed-End Growth

Shashank Shekhar and Marie-France Carlier

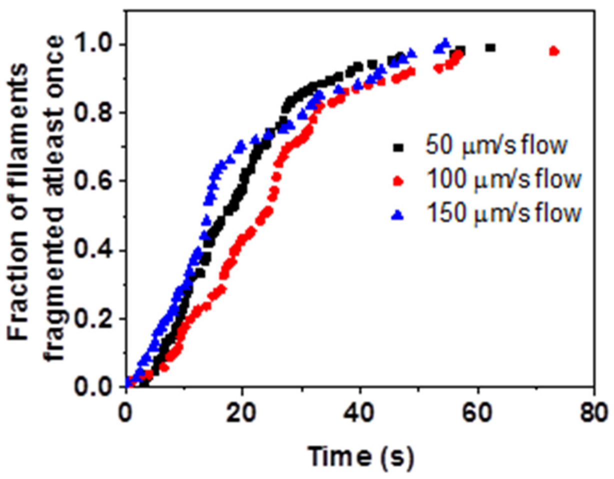

Figure S1. ADF-induced fragmentation is not affected by flow (in range of flow-rates used here). Related to Figure 1.

Frequency of ADF-induced fragmentation (at 5 µM ADF) was seen to be independent of the flow-rate in the range 50 µM/s to 150 µM/s flows (n = 100 filaments). ADF-induced depolymerization and fragmentation measurements were carried out at around 50 µM/s flow rates.

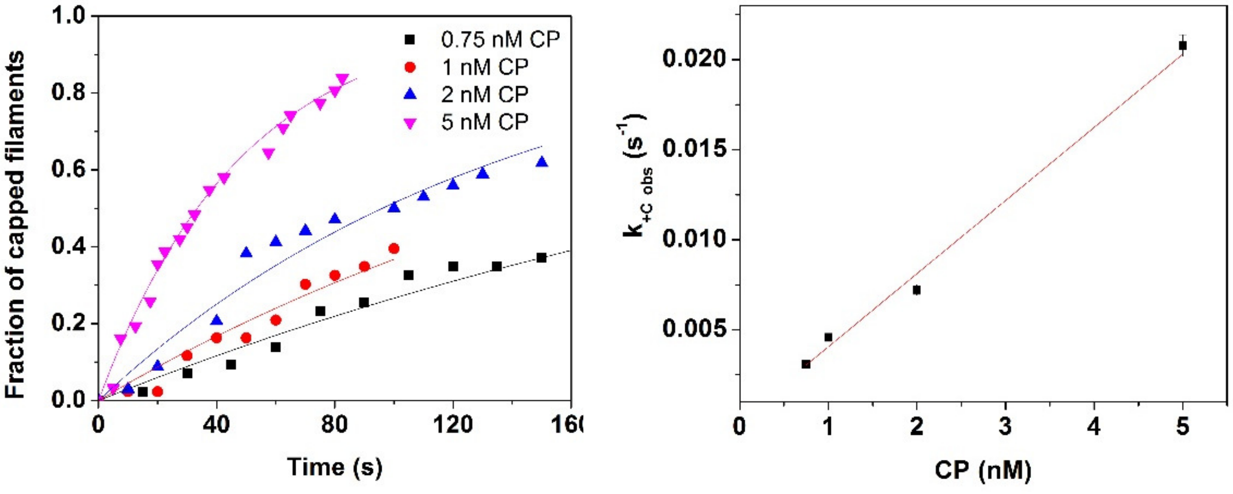

A

B

Figure S2. Measurement of the association rate constant of CP to the barbed end (k_{+C}). Related to Figure 3.

(A) Measurement of association rate constant for CP for tracer barbed end. Increase in fraction of barbed ends being capped by Capping Protein (B + C → BC) in the presence of 0.75 nM (black), 1 nM (red), 2 nM (blue) and 5 nM (magenta) CP. Symbols are actual data and lines are exponential fits (n = 30-40 filaments). (**B**) Linear fit of the relationship between observed on-rate of CP on CP concentration gives k_{+C} = 4.06± 0.17 µM^{-1}.s^{-1}.
